# Exploring the association between bullying and depression symptoms among adolescents in Bangladesh: Findings from nationally representative survey

**DOI:** 10.1371/journal.pmen.0000722

**Published:** 2026-10-28

**Authors:** Ruhul Amin, A S M Rafad Asgar

**Affiliations:** Bangladesh Institute of Governance and Management (BIGM), Dhaka, Bangladesh; FSBSI Scientific Research Institute of Neurosciences and Medicine: FGBNU Naucno-issledovatel'skij institut nejronauk i mediciny, RUSSIAN FEDERATION

## Abstract

Bullying victimization is an important public health concern that has been linked to adverse psychological outcomes among adolescents. The study used mental health well-being data collected in the most recent Bangladesh Adolescent Health and Wellbeing Survey (2019–2020) (BAHWS). A total of 9,116 adolescents aged 15–19 years with complete depression data were included in the analysis. A three-level linear mixed-effects model was employed to account for the hierarchical structure of the survey, with adolescents nested within primary sampling units and districts. Additional analyses examined gender and marital status interactions, subgroup differences, and the cumulative association between multiple forms of bullying and depressive symptoms using a composite bullying measure. The findings revealed that verbal bullying (β = 1.39, p < 0.001) and cyberbullying (β = 1.15, p = 0.012) were independently associated with higher depressive symptom scores. The association between physical bullying and depressive symptoms differed significantly by gender, whereas interactions of gender with verbal and cyberbullying were not statistically significant. Exploratory subgroup analyses showed variation in the magnitude of estimated associations across ever-married females, unmarried females, and unmarried males. A graded association was observed for cumulative bullying exposure: compared with adolescents reporting no bullying exposure, those exposed to on (β = 1.25), two (β = 2.61), and three (β = 4.99) bullying types had progressively higher depressive symptom scores (all p < 0.001). Adolescent Age, Current school or college attendance and higher household wealth were associated with lower depressive symptom scores. Bullying victimization, particularly verbal bullying, cyberbullying, and exposure to multiple forms of bullying, was associated with higher depressive symptoms among Bangladeshi adolescents. These findings highlight the need for comprehensive bullying prevention strategies and accessible psychosocial support services, with particular attention to adolescents experiencing multiple forms of bullying and other vulnerable groups.

## Introduction

Over 20% of the population in Bangladesh are adolescents, making them a crucial demographic group for the nation's growth [1]. Adolescents, defined by the World Health Organization (WHO) as individuals aged 10–19, are at a critical juncture in their development, undergoing significant emotional, cognitive, and social changes. Mental health issues, particularly depression, pose a serious threat to their well-being, impacting their social interactions, emotional stability, and cognitive skills.

Depression is one of the leading mental health disorders affecting adolescents worldwide, with approximately one in seven adolescents experiencing depression and chronic major depression affecting approximately 2.7% of the global population, accounting for more than one-quarter of all major depressive disorder cases [2,3]. The prevalence of adolescent depression varies considerably across countries but remains consistently high. In high-income countries, the prevalence of adolescent depression has increased over time, accompanied by persistent gaps in access to mental health care [4–6]. Similarly, studies from low- and middle-income countries have reported a substantial burden of depressive symptoms among adolescents, including prevalence estimates of 20–25% in sub-Saharan Africa, 26.2% in China, and 53.2% among students in Saudi Arabia [7–10]. Collectively, these findings highlight adolescent depression as a major global public health concern that warrants effective prevention and intervention strategies.

In Bangladesh, the situation is particularly concerning, with an estimated 24.5% of adolescents experiencing depression. The prevalence is notably higher among females (27.0%) compared to males (22.0%) [7]. The impact of depression extends beyond emotional distress, disrupting adolescents’ academic performance and long-term life outcomes [8]. Alarmingly, over 80% of affected adolescents in Bangladesh do not seek professional help due to factors such as stigma, limited resources, and the lack of adolescent-friendly mental health services [9]. Addressing these barriers is essential to safeguard the depression and overall development of this vulnerable population.

Bullying, in its various forms, including physical bullying, verbal bullying, and cyberbullying, significantly exacerbates mental health problems among adolescents. Research shows that adolescents who experience bullying are more likely to report mental health issues such as anxiety, depression, and low self-esteem [10]. Bullying encompasses physical assault, verbal abuse, neglect, and other harmful behaviors, all of which can contribute to psychological distress and long-term consequences such as social disengagement and emotional instability [3,11]. Physical bullying, such as assault, hitting, pushing, or other forms of bodily harm, often leads to aggression and diminished self-worth [3,11,12]. Verbal bullying, which includes verbal abuse, neglect, and threats, has severe emotional and psychological repercussions [13]. Verbal bullying, in particular, has severe emotional and psychological repercussions, while physical bullying often leads to aggression and diminished self-worth [13]. Cyberbullying, a more recent concern, has been rising with increased digital connectivity, particularly affecting adolescents in urban areas [14].

Bullying victimization, defined as repeated exposure to intentional aggressive behavior within a relationship characterized by a power imbalance, is a common experience among adolescents and an important risk factor for depressive symptoms and other adverse psychological outcomes [15]. Evidence from South Asia indicates a high prevalence of bullying victimization, with 41% of adolescents in Pakistan reporting bullying experiences and similarly high levels of bullying among school-going girl adolescents in India[16,17]. Although the relative impact of different bullying forms varies across settings, studies have shown that both traditional bullying (e.g., physical bullying) and non-physical forms, including relational and cyberbullying, are associated with poorer depression symptoms outcomes among adolescents [18,19].

In Bangladesh, adolescence is shaped by socioeconomic and sociocultural circumstances that may influence both exposure to bullying and vulnerability to depressive symptoms. Marriage during adolescence remains an important social and public health concern in Bangladesh and occurs within a broader context of socioeconomic disadvantage, educational discontinuity, family expectations, and gender norms [20,21]. Early marriage in Bangladesh occurs within a broader context of socioeconomic disadvantage, educational discontinuity, family expectations, and gender norms. The Bangladesh Adolescent Health and Wellbeing Survey (BAHWS) 2019–20 illustrates these differences among adolescents aged 15–19: more than three-fourths of ever-married female adolescents lived in rural areas, only 25% had completed secondary education or higher compared with 35% of unmarried females, and 83% had dropped out of school compared with 15% of unmarried females [22]. Moreover, two-thirds of married female adolescents reported that they would have preferred to marry later than they actually did [22]. These findings suggest that adolescent marriage is embedded within broader educational, familial, socioeconomic, and gender-related circumstances rather than representing an isolated demographic characteristic. Such circumstances may also shape adolescents’ social relationships, exposure to interpersonal victimization, and vulnerability to depressive symptoms. Accordingly, marital status in the present study is considered an important social-contextual characteristic when examining the association between bullying victimization and depressive symptoms.

A study on bullying victimization and depressive symptoms in Bangladeshi adolescents is scarce, despite increasing acknowledgment of bullying as a significant public health issue. Structural and sociocultural factors, including gender inequality and socioeconomic disadvantage, may increase adolescents’ vulnerability to both bullying victimization and depressive symptoms [9]. Studies from Bangladesh have reported that 43% of adolescent girls in rural areas experienced bullying while travelling to and from school and that approximately 32% of adolescents reported experiences of cyberbullying [19,23]. Although previous studies have examined specific forms of bullying, violence, or cyberbullying in relation to depression or other adverse psychological outcomes, the available evidence remains fragmented and has largely focused on individual bullying experiences [7,14,18]. Furthermore, little is known about the relative associations of physical bullying, verbal bullying, and cyberbullying with depressive symptoms in a nationally representative sample or whether these associations vary based on adolescents’ sex and marital status.

The BAHWS 2019–2020 has a hierarchical data structure, with adolescents clustered within primary sampling units (PSUs) and districts [22]. Such clustering may introduce within-group correlation and contextual variation in depressive symptoms, making multilevel modelling appropriate for accounting for the non-independence of observations. Previous studies in Bangladesh examining bullying, depression, or related psychological outcomes have generally not explicitly accounted for this hierarchical structure or assessed contextual variation across geographic levels [7,18,19,23].

Therefore, this study aimed to examine the associations between physical bullying, verbal bullying, and cyberbullying and depressive symptoms among Bangladeshi adolescents using nationally representative data. To appropriately account for the survey's hierarchical structure, a three-level linear mixed-effects model was employed to estimate these associations while accounting for variation at the cluster and district levels. To address the study objective of examining the associations between physical bullying, verbal bullying, and cyberbullying and depressive symptoms among Bangladeshi adolescents, the following research hypotheses were formulated:

Hypothesis 1: Forms of bullying (physical bullying, verbal bullying, and cyberbullying) are associated with adolescent depression symptoms.

Hypothesis 2: The associations between different forms of bullying and depressive symptoms vary across three adolescent groups: ever-married females, unmarried females, and unmarried males.

Hypothesis 3: Exposure to a greater number of bullying types is associated with progressively higher depressive symptom scores among adolescents.

## Method

### Ethics statements

The institutional review board (IRB) of the International Center for Diarrhea Disease Research, Bangladesh (icddr,b) and MEASURE Evaluation/Data for Impact (D4I), University of North Carolina at Chapel Hill, USA, and the United States Agency for International Development (USAID)/Bangladesh determined that this study meets the definition of non-human subject research and exempted it from IRB oversight. Participants (Aged 15–19) provided verbal informed consent to participate in the survey.

### Data sources

The Mental Health data of the adolescents (15–19 years) in the study were extracted from the nationally representative Bangladesh Adolescent Health and Wellbeing Survey (BAHWS) 2019–2020 [22]. The survey employed a two-stage stratified sampling technique: at the first stage, primary sampling units (PSUs) were selected using the probability proportional to size (PPS) method, based on household numbers reported in the 2011 Population and Housing Census. The selected PSUs were categorized into Type I and Type II clusters to administer different questionnaire modules. In the second stage, 100 households were systematically selected from each sampled PSU.

A total of 19,888 adolescents aged 15–19 years were identified, including 5,066 ever-married females, 8,284 unmarried females, and 6,538 unmarried males. Marital status was defined according to the BAHWS classification. Eligible adolescents were identified through household screening and categorized as ever-married females, unmarried females, or unmarried males, with separate questionnaires administered to each group. In Bangladesh, the statutory minimum age for marriage is 18 years for females and 21 years for males. The Child Marriage Restraint Act, 2017 includes a special provision permitting marriage below the statutory age under specified circumstances, including consideration of the minor’s best interests, court direction, and parental or guardian consent [24]. The BAHWS did not collect information on the legal registration or procedures through which individual adolescent marriages occurred, including whether marriages below the statutory age followed this provision. Therefore, “ever-married” represents the survey-reported marital-status classification and should not be interpreted as verification of the legal procedure or statutory compliance of individual marriages.

The outcome variable in this study was derived from the PHQ-9 questionnaire. Only adolescents with complete and valid responses to all PHQ-9 items required to calculate the depression score were eligible for inclusion in the present analysis. Consequently, 9,116 adolescents had complete outcome data and were included in the analytical sample (S1 Fig). Missing information on the outcome variable was primarily attributable to incomplete responses to one or more PHQ-9 items. Missing values in covariates were handled using complete-case analysis; observations with missing covariate information were excluded only from the corresponding regression models. Because the primary exclusions were due to missing outcome data, the analytical sample was determined before model fitting. Additionally, we address the STROBE cross-sectional reporting guidelines [25] in the S1 Checklist.

### Outcome variables

Depressive symptoms were measured using a modified version of the Patient Health Questionnaire (PHQ)-9, a self-reported questionnaire consisting of 9 items assessing symptoms of depression during the previous two weeks [26]. The adolescent-modified PHQ-9 adapts question wording and order to improve suitability for teenagers and includes additional items related to functional impairment, suicidal thoughts, and prior suicide attempts. PHQ-9 items were recorded on a four-point Likert scale ranging from 0 (not at all) to 3 (nearly every day). Summing the 9 items yields a total score ranging from 0 to 27, with higher scores indicating greater depressive symptom severity, ranging from minimal to severe depression [27]. Previous studies have demonstrated acceptable psychometric properties of the adolescent-modified PHQ-9, including good internal consistency and validity among adolescents. In Bangladesh, the Bangla-adapted PHQ-9 underwent formal translation and cultural adaptation using forward–backward translation and pilot testing and demonstrated good reliability and validity (Cronbach’s α = 0.837) [28]. Additionally, the use of the PHQ-9 among Bangladeshi student populations has demonstrated excellent internal consistency and convergent validity [19].

The explanatory variables of the study comprised adolescents’ socioeconomic and demographic characteristics as well as experiences of bullying. These variables were organized into two main subsections: socioeconomic and demographic factors and bullying experiences.

### Socioeconomic and demographic variables

Socioeconomic and demographic characteristics included adolescents’ age (15, 16, 17, 18, and 19 years), place of residence (rural and urban), household wealth quintile (poorest, poorer, middle, richer, and richest), gender, and current school or college attendance status (yes/no). Marital status was based on the classification used in the BAHWS 2019–20, which categorized female adolescents aged 15–19 as ever-married or unmarried. Among the ever-married female adolescents surveyed, 97% were currently married [18]. Only unmarried male adolescents were included in the BAHWS; therefore, marital-status comparisons in the present analysis were restricted to the adolescent groups represented in the survey. Also, a binary variable was created to indicate internet use, coded as “Yes” if the adolescent had access to a smartphone with internet connectivity or a computer with internet access, and “No” otherwise. These variables were included to capture key structural and individual-level determinants associated with adolescent mental health outcomes.

### Bullying experiences

Bullying experiences were assessed across three major domains of violence exposure during the 12 months preceding the survey, including physical violence, verbal/social bullying, and cyberbullying. Physical bullying was measured by asking respondents whether, in the past 12 months, anyone had slapped, pushed, or pulled their hair; punched, thrown something at them, or hit them with a stick or other object; kicked, dragged, or beaten them; attempted to choke them or deliberately burn them using fire, hot objects, or acid; or threatened or attacked them with a knife, gun, or any other weapon. A response of “yes” to any of these items was considered exposure to physical violence. Verbal bullying was assessed by asking whether, in the past 12 months, anyone had cursed them, made insulting comments, or called them names; spread lies or false accusations; issued written or verbal threats; or excluded them socially, such as not allowing participation in play, conversations, or peer interactions. A positive response to any item indicated exposure to verbal/social bullying. Cyberbullying was measured by asking whether, during the past 12 months, anyone had used a mobile phone or the internet to harass, intimidate, or spread negative content or images about them without consent. Respondents who reported exposure were further asked about the frequency of occurrence (once, two to four times, or five or more times). It is important to note that bullying exposures were assessed over a 12-month recall period, whereas depressive symptoms were measured over the preceding two weeks. This difference in recall periods should be considered when interpreting the findings, as it may limit temporal alignment between exposure and outcome and restrict causal inference [22]. The BAHWS measures of violence and bullying were not restricted to peer-perpetrated experiences. For respondents reporting exposure, the survey collected information on perpetrators, including parents, spouses, in-laws, other family members, teachers, friends/classmates, neighbors, unknown persons, and others. Consequently, among ever-married female adolescents, some reported experiences, particularly physical violence and verbal/social bullying, could have occurred within marital or household relationships. In this study, exposures were retained according to the original BAHWS operational definitions and were not reclassified according to perpetrator. Therefore, the term “bullying” refers to the survey-defined exposure to the specified acts and should not be interpreted exclusively as peer bullying. To assess the cumulative association of bullying victimization with depressive symptoms, a composite bullying variable (none, one, two, or three bullying types) was included in an additional multilevel linear mixed-effects model.

### Statistical analysis

SAS version 9.4 was used for data cleaning, recoding and analysis. Descriptive statistics (frequencies, percentages, and means) of each selected covariate were performed. We also performed spatial mapping using the ggplot2 packages in the R programming language version 4.5.1 [29]. T-tests and analysis of variance (ANOVA) were used to assess the relationship between different explanatory variables with depression at a 95% confidence interval (CI). To ensure the survey’s representativeness, sample weights were applied. The BAHWS 2019–20 data were collected through a multi-stage cluster sampling design, where adolescents are nested within clusters, which are further nested within district levels [22]. Since the study employed a multilevel data structure, we utilized a linear mixed model to identify significant determinants while accounting for within-group correlation [30,31]. The linear mixed model, also known as a multilevel model, is an extension of the linear model where the random effects of hierarchical levels (such as villages and regions where an individual belongs to) are considered in addition to the fixed effects of factors and covariates, which were assumed to have a linear relationship with the dependent variable [32,33]. In this study, we employed a three-level mixed-effects regression model. To account for this hierarchical structure, we fitted three-level linear mixed-effects models with adolescents (level 1) nested within PSUs (level 2), which were nested within districts (level 3).

Let $y_{ijk}$ denotes the depression (a continuous response variable) for the $k^{th}$ adolescents belong to $j^{th}$ cluster (PSU) within $i^{th}$ region (district). The three-level random-intercept model was specified as


$$\begin{array}{c} y_{ijk}=x_{ijk}^{T}\beta +\lambda_{i}+\eta_{ij}+\varepsilon_{ijk} \end{array}$$

where, $x_{ijk}^{T}\;$ is a vector of explanatory information, β is the vector of the regression parameters and $\lambda_{i}$, $\eta_{ij}$ and $\varepsilon_{ijk}$ are districts, PSU, and adolescent-specific random effects, respectively. It was assumed that the level-specific random effects were identically and independently distributed with mean zero and homoscedastic error variances $\sigma_{\lambda }^{\text{2}}$, $\sigma_{\eta }^{\text{2}}$ and $\sigma_{\varepsilon }^{\text{2}}$ respectively [31]. Assuming normality of the level-specific random effects, the model can be developed following the weighted maximum likelihood (ML) method [30]. The fitted models can be used to estimate mean and distribution functions of mental well-being at different hierarchies (for example, PSU and region in this study) [31].

To account for the complex survey design and unequal probabilities of selection, the individual sampling weight $\omega_{ijk}$ provided in the BAHWS dataset was incorporated during model estimation. Specifically, the model parameters were estimated by maximizing a weighted likelihood, where each adolescent’s contribution to the likelihood was weighted by $\omega_{ijk}:$


$$\begin{array}{c} L_{\omega }(\theta )=\;\prod_{i=\text{1}}^{I}\prod_{j=\text{1}}^{J_{i}}\prod_{k=\text{1}}^{n_{ij}}{\left[L_{ijk}(\theta )\right]}^{\omega_{ijk}} \end{array}$$

or equivalently,


$$\begin{array}{c} l_{\omega }(\theta )=\;\sum_{i=\text{1}}^{I}\sum_{j=\text{1}}^{J_{i}}\sum_{k=\text{1}}^{n_{ij}}\omega_{ijk}\;l_{ijk}(\theta ) \end{array}$$

Where $L_{\omega }(\theta$and $l_{\omega }(\theta )$ denote the likelihood and log-likelihood contribution of $k^{th}$ adolescents belong to $j^{th}$ cluster (PSU) within $i^{th}$ region (district) and represents all fixed-effect and variance parameters. In SAS PROC MIXED, this weighting was implemented using the WEIGHT statement, while district- and PSU-level clustering was modeled through random intercepts [34]. Model assumptions were evaluated by examining residual-versus-predicted plots, and normal Q-Q plot. To determine the appropriate multilevel structure, unconditional two-level and three-level linear mixed-effects models were initially fitted and compared using Akaike’s Information Criterion (AIC), conditional AIC (AICc), Bayesian Information Criterion (BIC), Inter cluster correlation (ICC) and log-likelihood ratio test [31,35]. The model providing better overall fit based on these criteria was retained for the subsequent adjusted analyses. Risk factors of adolescents’ depression scores were examined by the likelihood ratio-based Type-III test. The Type-III test of a predictor of interest is the joint test in which the parameters associated with the predictor were zero [32].

Following selection of the multilevel structure, three sets of adjusted linear mixed-effects analyses were conducted. First, the primary model was fitted to the total sample, with physical bullying, verbal bullying, and cyberbullying entered simultaneously together with the prespecified sociodemographic covariates. Interaction terms between each bullying type and gender and between each bullying type and marital status were included to formally assess potential effect modification. Second, separate multivariable models were fitted for the three adolescent subgroups represented in the BAHWS: ever-married females, unmarried females, and unmarried males. Physical bullying, verbal bullying, and cyberbullying were entered simultaneously with the relevant covariates in each subgroup model. Because married adolescent males were not represented in the BAHWS, subgroup analyses were restricted to these three groups. These analyses were considered exploratory and were used to describe the magnitude and direction of associations within each subgroup; formal evidence of between-group heterogeneity was assessed using the interaction terms in the total-sample model. Subgroup-specific estimates were summarized using a forest plot. Finally, an additional model was fitted to the total sample using the composite bullying measure as a categorical exposure with four levels (none, one, two, or three bullying types), with no bullying exposure as the reference category. The coefficients for one, two, and three bullying types represent the adjusted mean differences in depressive symptom scores relative to adolescents reporting no bullying exposure, after adjustment for the prespecified covariates and the multilevel structure of the data. All adjusted models incorporated sampling weights and random intercepts for PSUs and districts.

## Results

### Descriptive characteristics and distribution of depressive symptoms

Among the 9,116 adolescents included in the study, the largest proportion were aged 16 years (22.83%), followed by 15 years (20.81%), 17 years (20.74%), and 18 years (19.77%), while 19-year-olds constituted the smallest age group (15.87%). The majority of participants resided in rural areas (75.30%), whereas 24.70% lived in urban settings. Most adolescents were unmarried (72.75%), whereas 27.25% were ever-married. Females represented a substantially larger share of the sample (69.49%) than males (30.51%). Regarding gender–marital status, unmarried females comprised the largest subgroup (42.25%), followed by unmarried males (30.51%) and married females (27.25%). Approximately 29.11% of adolescents reported using an internet-enabled device, while 70.89% did not. The distribution across wealth quantiles was relatively balanced, with the highest proportion belonging to the middle wealth group (21.93%), followed by the richer (21.47%), richest (20.18%), poorer (19.51%), and poorest (16.91%) categories. Nearly two-thirds of adolescents (62.56%) were currently attending school or college, whereas 37.44% were not.

Verbal bullying was the most frequently reported form of bullying, affecting 31.64% of adolescents, followed by physical bullying (20.79%) and cyberbullying (6.60%). When bullying experiences were combined into a composite measure, 60.47% of adolescents reported no bullying exposure, 22.45% experienced one type of bullying, 14.66% experienced two types, and 2.42% reported exposure to all three forms of bullying. (Table 1). The prevalence of bullying varied across different divisions. Barisal division had the highest incidence of physical bullying, verbal bullying, and cyberbullying, respectively, among adolescents (S2 Fig). The distributions of key sociodemographic characteristics were broadly similar between the included and excluded adolescents (S1 Table).

**Table 1:** Demographic Profile of the Study.

Variable Name	Attribute	Frequency (%)	Weighted Percentage
Age	15	1902(20.86)	20.81
	16	2037(22.35)	22.83
	17	1914(21)	20.74
	18	1817(19.93)	19.77
	19	1446(15.86)	15.87
Residence	Urban	2074(22.75)	24.7
	Rural	7042(77.25)	75.30
Marital Status	Yes	2470(27.10)	27.25
	No	6646(72.90)	72.75
Gender	Male	2764(30.32)	30.51
	Female	6352(69.68)	69.49
Gender–Marital types	Married Female	2470(27.10)	27.25
	Unmarried Female	3882(42.58)	42.25
	Unmarried Male	2764(30.32)	30.51
Use Internet Device	No	6516(71.48)	70.89
	Yes	2600(28.52)	29.11
Wealth Quantile	Poorest	1559(17.1)	16.91
	Poorer	1765(19.36)	19.51
	Middle	2043(22.41)	21.93
	Richer	1955(21.45)	21.47
	Richest	1794(19.68)	20.18
Currently Attend School / College	No	3394(37.82)	37.44
	Yes	5579(62.18)	62.56
Physical bullying	No	5513(79.92)	79.21
	Yes	1385(20.08)	20.79
Verbal bullying	No	4727(68.53)	68.36
	Yes	2171(31.47)	31.64
Cyber bullying	No	6453(93.55)	93.40
	Yes	445(6.45)	6.60
Composite bullying	None	4194(60.80)	60.47
	One Type	1564(22.67)	22.45
	Two Type	983(14.25)	14.66
	Three Type	157(2.28)	2.42
Division	Barisal	559(6.13)	6.30
	Chittagong	2105(23.09)	19.34
	Dhaka	1805(19.8)	24.22
	Khulna	886(9.72)	10.85
	Mymensingh	649(7.12)	7.46
	Rajshahi	1133(12.43)	12.14
	Rangpur	1137(12.47)	12.08
	Sylhet	842(9.24)	7.62

Table 2 presents the mean adolescent mental health score across different socio-demographic and bullying-related characteristics. Mental health scores increased progressively with age, rising from 4.28 among 15-year-olds to 5.19 among 19-year-olds, indicating poorer mental health among older adolescents. Urban adolescents reported slightly higher mean mental health scores (4.83) than their rural counterparts (4.67). Significant differences were also observed by marital status and gender. Married adolescents exhibited higher mean scores (5.58) compared to unmarried adolescents (4.38), while females reported substantially higher scores (5.22) than males (3.55). When gender and marital status were combined, married females had the highest mean mental health score (5.58), followed by unmarried females (4.98), whereas unmarried males reported the lowest score (3.55). Internet device use was not significantly associated with mental health scores, although adolescents who used internet-enabled devices reported a slightly lower mean score (4.63) than non-users (4.74). Differences across wealth quantiles were modest and statistically non-significant, with the highest mean score observed among adolescents in the richest wealth group (4.62), followed by the poorest group (4.51). Consistent with the protective role of education, adolescents not attending school or college had significantly higher mental health scores (5.24) compared with those currently attending educational institutions (4.38). Adolescents exposed to cyberbullying reported the highest mean score (7.44), followed by physical bullying (6.28) and verbal bullying (6.08), whereas those not experiencing these forms of bullying had substantially lower scores. A clear dose–response relationship was observed for the composite bullying measure: adolescents reporting no bullying exposure had the lowest mean score (3.97), while those experiencing one type (5.11), two types (6.48), and all three types of bullying (9.24) showed progressively higher scores. Significant regional differences were also evident, with adolescents from Barisal Division reporting the highest mean mental health score (5.36), followed by Sylhet (5.03) and Rangpur (4.97), whereas those from Dhaka Division reported the lowest mean score (4.47) (Table 2). The distribution of the depression category with bullying is provided in S3 Fig.

**Table 2:** Differences in mean effect on adolescent mental health.

Variable	Attribute	Mean (Std. Dev)	P-value
Age	15	4.28(3.55)	<0.001
	16	4.47(3.58)	
	17	4.89(3.89)	
	18	4.86(3.82)	
	19	5.19(4.01)	
Residence	Urban	4.83(3.92)	0.003
	Rural	4.67(3.72)	
Marital Status	No	4.38(3.53)	<0.001
	Yes	5.58(4.09)	
Gender	Male	3.55(3.26)	<0.001
	Female	5.22(3.87)	
Gender–Marital types	Married Female	5.58(4.09)	<0.001
	Unmarried Female	4.98(3.69)	
	Unmarried Male	3.55(3.26)	
Use Internet Device	No	4.74(3.75)	0.203
	Yes	4.63(3.83)	
Wealth Quantile	Poorest	4.51(3.69)	0.115
	Poorer	4.22(3.51)	
	Middle	4.24(3.54)	
	Richer	4.35(3.48)	
	Richest	4.62(3.72)	
Currently Attend School /college	No	5.24(3.97)	<0.001
	Yes	4.38(3.59)	
Physical bullying	No	4.31(3.53)	<0.001
	Yes	6.28(4.37)	
Verbal bullying	No	4.09(3.52)	<0.001
	Yes	6.08(4.02)	
Cyber bulling	No	4.53(3.69)	<0.001
	Yes	7.44(4.27)	
Composite bulling	None	3.97(3.65)	<0.001
	One Type	5.11(3.65)	
	Two Type	6.48(4.09)	
	Three Type	9.24(4.57)	
Division	Barisal	5.36(3.87)	<0.001
	Chittagong	4.65(3.48)	
	Dhaka	4.47(4.29)	
	Khulna	4.76(3.83)	
	Mymensingh	4.50(3.27)	
	Rajshahi	4.73(3.62)	
	Rangpur	4.97(3.69)	
	Sylhet	5.03(4.01)	

### Selection of the best-fitting multilevel model

Table 3 compares the null two-level and three-level mixed-effects models. The null three-level model partitioned variance into district (0.22) and cluster (0.48) components and demonstrated better model fit than the two-level model, as indicated by lower AIC, AICC, BIC, and −2 log-likelihood values. The ICC increased slightly from 5.10% to 5.18%, suggesting a modest gain in explained variance. The likelihood ratio test showed that the null three-level model fit the data significantly better than the two-level model (χ² = 19.16, p < 0.001). Visual inspection indicated no substantial departures from normality and homoscedasticity, supporting the adequacy of the fitted linear mixed-effects models (S4 Fig).

**Table 3:** Summary statistics of the fitted null multilevel model for depression.

	Null Two-Level Model	Null Three-Level Model
**Variance Component**		
District Variance ($\sigma_{d}^{\text{2}}$)	–	0.22
Cluster Variance ($\sigma_{c}^{\text{2}}$)	0.69	0.48
Residual Variance ($\sigma_{r}^{\text{2}}$)	13.52	13.52
**Model Fit Statistics**		
AIC	50762.1	50744.9
AICC	50762.1	50744.9
BIC	50773.8	50753.5
ICC*100	5.10%	5.18%
−2 Log Likelihood	50756.1	50736.9
LR test of *H*_0_: $\sigma_{\vartheta }^{\text{2}}$ = 0, *χ*^2^ = 19.16, *p* value = < 0.001 (2-level vs 3-level)		

### Multivariable associations of bullying and sociodemographic factors with depressive symptoms

Table 4 presents the adjusted regression coefficients from the multivariable three-level linear mixed-effects model examining the associations between bullying victimization and depressive symptoms among Bangladeshi adolescents. All three forms of bullying were significantly associated with higher depressive symptom scores. Adolescents who experienced physical bullying had significantly higher depression scores than those who did not (β = 0.64, SE = 0.19, p < 0.001). Similarly, verbal bullying (β = 1.39, SE = 0.18, p < 0.001) and cyberbullying (β = 1.15, SE = 0.46, p = 0.012) were positively associated with depressive symptoms.

**Table 4:** Associated factors for adolescent depression.

Variable	Attribute	Adjusted Coefficient (SE)	P-value
Physical Bullying	Yes	0.64(0.19)	<0.001
	No(ref)	.	.
Verbal Bullying	Yes	1.39(0.18)	<0.001
	No(ref)		
Cyber Bullying	Yes	1.15(0.46)	0.012
	No(ref)	.	.
Gender	Female	1.43(0.13)	<0.001
	Male(ref)		
Marital Status	Yes	0.05(0.13)	0.682
	No(ref)		
Physical Bullying*Gender		1.12(0.26)	<0.001
Verbal Bullying*Gender		-0.20(0.24)	0.408
Cyber Bullying*Gender		0.74(0.51)	0.153
Physical Bullying*Marital Status		-0.42(0.29)	0.145
Verbal Bullying*Marital Status		0.35(0.25)	0.165
Cyber Bullying*Marital Status		-0.13(0.39)	0.743
Age	16	0.03(0.13)	0.761
	17	0.41(0.13)	0.002
	18	0.36(0.14)	0.009
	19	0.72(0.15)	<0.001
	15 (ref)	.	.
Residence	Rural	-0.15(0.14)	0.257
	Urban(ref)	.	.
Use Internet Device	Yes	0.22(0.10)	0.027
	No(ref)		
Wealth Quantile	Poorer	-0.35(0.14)	0.013
	Middle	-0.42(0.14)	0.003
	Richer	-0.41(0.15)	0.005
	Richest	-0.36(0.16)	0.028
	Poorest(ref)	.	.
Currently attending school/college	Yes	-0.43(0.11)	<0.001
	No(ref)	.	.
Division	Chittagong	-0.07 (0.33)	0.816
	Dhaka	-0.34 (0.30)	0.253
	Khulna	-0.12 (0.32)	0.714
	Rajshahi	-0.31 (0.32)	0.341
	Rangpur	-0.03 (0.33)	0.917
	Sylhet	0.34(0.38)	0.371
	Mymensingh	-0.06 (0.38)	0.853
	Barisal(ref)	.	.

Female adolescents had significantly higher depressive symptom scores than male adolescents (β = 1.43, SE = 0.13, p < 0.001), whereas marital status was not independently associated with depressive symptoms after adjustment for other covariates (β = 0.05, SE = 0.13, p = 0.682). A significant interaction was observed between physical bullying and gender (β = 1.12, SE = 0.26, p < 0.001), indicating that the association between physical bullying and depressive symptoms differed by gender. However, the interaction terms for verbal bullying (β = −0.07, SE = 0.21, p = 0.759) and cyberbullying (β = 0.68, SE = 0.49, p = 0.167) were not statistically significant. The adjusted model contained no statistical evidence that the relationships between the different kinds of bullying and depressed symptoms differed depending on marital status (Table 4).

Depressive symptom scores increased with age, with adolescents aged 17 (β = 0.41, SE = 0.13, p = 0.002), 18 (β = 0.36, SE = 0.14, p = 0.009), and 19 years (β = 0.72, SE = 0.15, p < 0.001) reporting significantly higher scores than those aged 15 years. Adolescents from poorer (β = −0.35, SE = 0.14, p = 0.013), middle (β = −0.42, SE = 0.14, p = 0.003), richer (β = −0.41, SE = 0.15, p = 0.005), and richest households (β = −0.36, SE = 0.16, p = 0.028) had significantly lower depressive symptom scores than those from the poorest households. Adolescents currently attending school or college also had significantly lower depression scores (β = −0.43, SE = 0.11, p < 0.001), whereas those using internet-enabled devices had slightly higher depressive symptom scores (β = 0.22, SE = 0.10, p = 0.027). No statistically significant associations were observed for place of residence or administrative division. (Table 4)

### Subgroup-specific associations between bullying and depressive symptoms

The forest plot (Fig 1) reveals a significant relationship between bullying (physical, verbal, and cyber) and depression across three groups: married females, unmarried females, and unmarried males. For all forms of bullying, the coefficients were significantly positive, indicating an increase in depression for individuals who experienced bullying. Physical bullying was associated with increased depression across all groups, with the strongest associations observed among unmarried females (β ≈ 2.42) and married females (β ≈ 2.38), while the effect was comparatively smaller among unmarried males (β ≈ 1.32). Verbal bullying also showed a significant positive relationship with depression, with coefficients of approximately 2.28 for married females, 2.06 for unmarried females, and 1.69 for unmarried males. Cyberbullying exhibited the largest association with depression in all three groups. The effect was strongest among married females (β ≈ 2.62), followed closely by unmarried females (β ≈ 2.55), and remained substantial among unmarried males (β ≈ 2.30). Similarly, composite bullying was significantly associated with higher depression scores, with coefficients of approximately 2.18 among married females, 2.12 among unmarried females, and 1.49 among unmarried males.

**Fig 1:**
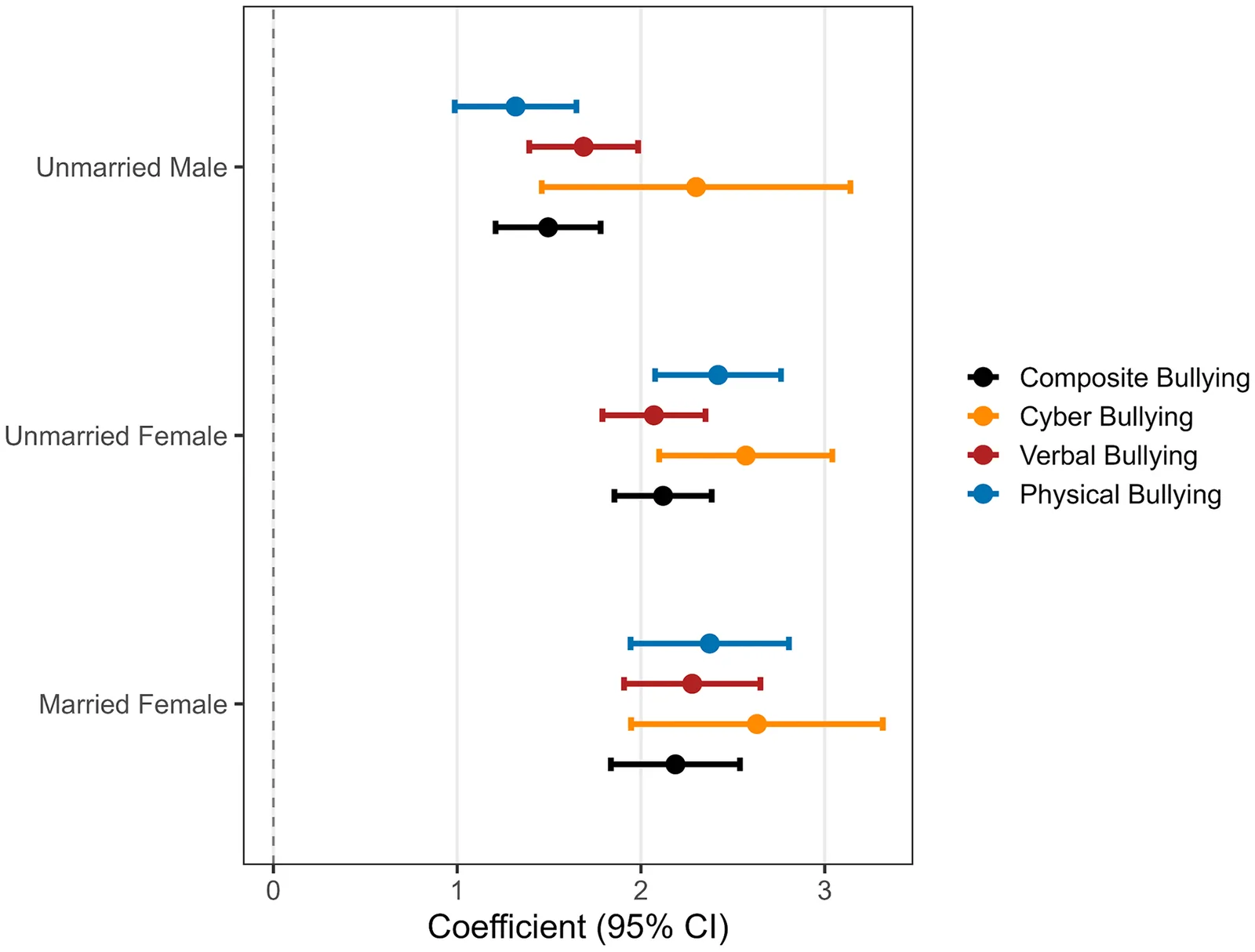
Forest Plot of Bullying Effect on Adolescents’ depression.

### Composite bullying and depressive symptoms

Table 5 shows the relationship between composite bullying and adolescent mental health. Compared to no bullying, exposure to one (β = 1.25, p < 0.001), two (β = 2.61, p < 0.001), and three types (β = 4.99, p < 0.001) of bullying was associated with progressively higher scores.

**Table 5:** Adjusted associations between composite bullying and depressive symptoms among Bangladeshi adolescents.

Composite Bullying	Coefficient (SE)	p-value
One	1.25 (0.10)	<0.001
Two	2.61 (0.12)	<0.001
Three	4.99(0.28)	<0.001
None (ref)	.	.

Note: Only coefficients for the composite bullying variable are presented. No bullying exposure was used as the reference category; therefore, the coefficients for one, two, and three bullying types represent adjusted mean differences in depressive symptom scores relative to adolescents reporting no bullying exposure. All estimates are adjusted for age, residence, school attendance, division, marital status, gender, internet device use, household wealth, and other covariates included in the model.

## Discussions

This study examined the associations between physical bullying, verbal bullying, and cyberbullying and depressive symptoms among Bangladeshi adolescents using nationally representative data while accounting for the hierarchical structure of the survey through a three-level linear mixed-effects model. Three principal findings emerged from the study. First, after taking into account sociodemographic and contextual characteristics, physical, verbal, and cyberbullying were each found to be associated with higher levels of depressive symptoms. Second, the interaction analyses showed that the link between physical bullying and depressive symptoms varied according to gender, while no significant gender-specific difference was found for either verbal or cyberbullying, and no significant effect modification by marital status was detected for any of the types of bullying. Third, the more types of bullying a person was exposed to, the higher their depressive symptom scores became, showing a cumulative association that increased in degree. Collectively, these findings underscore bullying victimization as an important correlate of depressive symptoms among Bangladeshi adolescents and highlight the need to consider both individual experiences and broader social contexts when developing prevention and intervention strategies.

The findings generally support Hypothesis 1, indicating that different forms of bullying are associated with depressive symptoms among Bangladeshi adolescents, although the magnitude of these associations varied across bullying types. After adjustment for sociodemographic and contextual factors, physical bullying, verbal bullying, and cyberbullying were all independently associated with higher depressive symptom scores. Furthermore, the significant interaction between physical bullying and gender suggests that the association between physical bullying and depressive symptoms differed between male and female adolescents. These findings are consistent with previous studies demonstrating that adolescents who experience bullying are more likely to report depressive symptoms and other adverse psychological outcomes than those who are not exposed to bullying [36–40]. Several mechanisms may explain these associations. Bullying victimization can undermine self-esteem, weaken peer relationships, and increase feelings of social isolation and helplessness, all of which have been linked to depressive symptoms during adolescence [41–43]. Among the different forms of bullying, verbal bullying showed the strongest observed association with depressive symptoms in the adjusted model, suggesting that repeated ridicule, humiliation, and verbal aggression may have substantial psychological consequences even in the absence of physical harm [13,44]. Cyberbullying was also independently associated with higher depressive symptom scores. Unlike traditional bullying, cyberbullying may occur at any time, rapidly reach a wide audience through digital platforms, and leave a persistent online presence, potentially prolonging emotional distress and increasing adolescents’ psychological vulnerability [45,46,38]. Overall, these findings provide further support for Hypothesis 1 and emphasize the importance of addressing physical, verbal, and cyberbullying as part of comprehensive strategies to reduce depressive symptoms among Bangladeshi adolescents.

Formal interaction analyses provided only partial evidence that the associations between bullying victimization and depressive symptoms varied by gender and marital status. In the adjusted multilevel model, female adolescents had significantly higher scores for depressive symptoms than males, but this main effect should be separated from the effect modification of the association between bullying and depression. A significant interaction with gender was found only in the case of physical bullying, which means that the link between physical bullying and depressive symptoms was different for male and female adolescents. On the other hand, the gender interactions for verbal and cyberbullying were not statistically significant, and none of the interactions between bullying and marital status reached significance. As has been suggested by earlier studies, the psychological effects of bullying might vary depending on gender and on broader social and cultural contexts, although the degree and type of these differences differ from one population to another [36–40,47–49]. Thus, the present findings provide evidence of gender-related heterogeneity specifically in the association between physical bullying and depressive symptoms, but not for verbal or cyberbullying or for effect modification by marital status.

Further, the subgroup analyses indicated that ever-married and unmarried female adolescents generally exhibited stronger associations between bullying victimization and depressive symptoms than unmarried male adolescents. These findings are consistent with evidence suggesting that adolescent girls in South Asian settings are more vulnerable to depressive symptoms because of gender inequality, early marriage, restrictive social norms, and unequal educational and social opportunities [39,40,49]. Marriage during adolescence may further increase psychological vulnerability by introducing additional social and familial responsibilities at a developmentally sensitive stage [50]. Accordingly, the subgroup findings should be regarded as exploratory and interpreted alongside the formal interaction analyses. Hypothesis 2 was partially supported: the association between physical bullying and depressive symptoms differed significantly by gender, whereas no significant gender-specific variation was observed for verbal or cyberbullying, and marital status did not significantly modify the association between any bullying type and depressive symptoms.

An important finding was the cumulative association between exposure to multiple forms of bullying and depressive symptoms. Compared with adolescents reporting no bullying, those exposed to one, two, and three types had progressively higher depressive symptom scores. This graded pattern is consistent with a dose–response association, suggesting that the psychological burden of bullying may increase as adolescents experience multiple forms of victimization. Exposure to overlapping forms of bullying may compound experiences of humiliation, fear, social exclusion, and reduced perceived support, thereby increasing vulnerability to depressive symptoms [41–43]. These findings support Hypothesis 3 and highlight the importance of identifying adolescents experiencing multiple forms of bullying and implementing comprehensive prevention and psychosocial support strategies.

Beyond bullying victimization, several sociodemographic characteristics were independently associated with depressive symptoms among Bangladeshi adolescents. Older adolescents experienced higher levels of depressive symptoms than younger adolescents, consistent with previous studies suggesting that increasing academic demands, identity formation, and social transitions during late adolescence contribute to greater psychological vulnerability [47,51,52]. In contrast, adolescents who were currently attending school or college had lower depression scores, supporting evidence that educational engagement provides structured social environments, peer support, and access to psychosocial resources that may protect against depressive symptoms [7,43].

Household wealth was also associated with depressive symptoms, although the relationship was not strictly linear. Compared with adolescents from the poorest households, those from poorer, middle, and richer households reported significantly lower depression scores, whereas no significant difference was observed for adolescents from the richest households. These findings suggest that socioeconomic disadvantage may increase vulnerability to depressive symptoms through material deprivation, financial insecurity, and reduced access to supportive resources [3,11,53]. Internet device use was associated with a modest increase in depressive symptom scores. Although this association does not establish that device use causes depression, it may reflect differences in digital exposure, including greater opportunities for online social comparison or adverse online experiences, and warrants further investigation. In contrast, place of residence and administrative division were not independently associated with depressive symptoms after adjustment for other covariates, suggesting that individual- and household-level characteristics may play a more prominent role than geographic location in explaining depressive symptoms among Bangladeshi adolescents.

### Strengths and limitations

This study has several important strengths. It utilized nationally representative data from the Bangladesh Adolescent Health and Wellbeing Survey, enhancing the generalizability of the findings to Bangladeshi adolescents. Depressive symptoms were assessed using a validated PHQ-9 instrument, and multiple forms of bullying were examined simultaneously within a single analytical framework. The application of a three-level linear mixed-effects model accounted for the hierarchical survey structure and enabled estimation of both individual- and contextual-level variation. Formal interaction tests were conducted for each bullying type by both gender and marital status, allowing potential effect modification to be evaluated directly rather than inferred from separate subgroup-specific statistical significance tests. In addition, the inclusion of a composite bullying measure demonstrated a clear dose–response relationship between cumulative bullying victimization and depressive symptoms, while formal interaction analyses provided further insight into gender differences in these associations. Collectively, these methodological strengths provide robust evidence to inform bullying prevention and adolescent mental health policies in Bangladesh.

This study is based on cross-sectional data, which limits the ability to establish causal relationships between bullying and depression. Self-reported measures may introduce recall bias, and the dataset lacked information on treatment-seeking practices. Another limitation is that the BAHWS did not include married adolescent boys [22]; therefore, subgroup analyses were restricted to ever-married females, unmarried females, and unmarried males. Additionally, the BAHWS did not document whether marriages involving adolescents below the statutory minimum age followed the legally prescribed special-circumstances procedure; therefore, the legal circumstances of individual marriages could not be verified. Moreover, BAHWS-defined bullying was not restricted to peer perpetrators. Among ever-married females, reported perpetrators included husbands, in-laws, and other family members; thus, some experiences may overlap with intimate partner or domestic violence and should not be interpreted exclusively as peer bullying. This potential overlap could not be disentangled in the present secondary analysis and should be considered when interpreting findings for ever-married females. Exclusion of adolescents with incomplete PHQ-9 data may also have introduced selection bias. Finally, bullying was assessed over the preceding 12 months, whereas depressive symptoms were measured over the preceding two weeks. Therefore, the findings should be interpreted as associations rather than temporal or causal effects. Future longitudinal studies with aligned assessment periods and clearer differentiation between peer bullying and marital or family violence are warranted.

## Conclusions

This nationally representative study demonstrates that physical, verbal, and cyberbullying are independently associated with higher depressive symptom scores among Bangladeshi adolescents. The association between physical bullying and depressive symptoms differed significantly by gender, whereas no evidence of gender-specific effect modification was observed for verbal or cyberbullying, and marital status did not significantly modify the association for any bullying type. Furthermore, exposure to an increasing number of bullying types was associated with progressively higher depressive symptom scores, highlighting the potential cumulative psychological burden of multiple forms of victimization.

These findings underscore the importance of comprehensive bullying-prevention strategies that address physical, verbal, and cyberbullying alongside accessible psychosocial support for adolescents. Particular attention should be given to adolescents experiencing multiple forms of bullying. Future longitudinal studies are needed to clarify the temporal relationship between bullying victimization and depressive symptoms and to further investigate how gender and other social contexts may shape these associations.

## Supporting information

S1 Table Excluded adolescent sociodemographic information. (DOCX)

S1 Fig Sample Size Selection. (TIF)

S2 Fig Division-wise prevalence of different types of bullying (a. Physical bullying; b. Cyberbullying; c. Verbal bullying; d. Ever bullied).
(TIF)

S3 Fig Depression Categories by Bullying Types. (TIF)

S4 Fig Diagnostic plots for assessing assumptions of the three-level linear mixed-effects model of depressive symptoms among Bangladeshi adolescents. ((A) Residuals versus predicted values for the three-level linear mixed-effects model. (B) Normal Q–Q plot of standardized residuals from the three-level linear mixed-effects model.).
(ZIP)

S1 Checklist STROBE Statement Checklist. (DOCX)
