# Supplementary material for: Exploring the association between bullying and depression symptoms among adolescents in Bangladesh: Findings from nationally representative survey
Source: PLOS Ment Health. 2026 Oct 28;3(10):e0000722. doi: 10.1371/journal.pmen.0000722 (PMC13716191; doi:10.1371/journal.pmen.0000722)
Supplement: Supplementary file 1 [file pmen.0000722.s007.pdf]

Ruhul Amin
Research Associate
Bangladesh Institute of Governance and Management
Dhaka-1207, Bangladesh
E-mail: ruhul.sta@gmail.com
ruhul.amin@bigm.edu.bd

Staff Editor
PLOS Global Public Health

Dear Professor Joanna Tindall, PhD

Please find the revised version of the manuscript (Submission ID: PGPH-D-25-01493) entitled: **"Exploring the Association Between Bullying and Mental Well-being Among Adolescents in Bangladesh: Findings from Nationally Representative Survey.**

We have revised the manuscript according to the reviewer's comments and suggestions.

Authors' Note: The major corrections and new addendum are highlighted in magenta color texts for EBM, red color texts for Reviewer in the revised manuscript. Additional corrections are highlighted in **a different color** following the journal template.

Legend (text colors for revision and corrections)

- EBM
- Reviewer

EBM

1. Please ensure that your Ethics Statement is available in its entirety at the beginning of your Methods section, under a subheading 'Ethics Statement'.

   Ans: We thank for pointing out this really important concern. We have addressed the issue by setting the Ethics Statement under the Methods section of the manuscript. The amendment (page 7, lines 177-183) states:

   "Ethics Statements
   The institutional review board (IRB) of the International Center

for Diarrhea Disease Research, Bangladesh (icddr,b) and MEASURE Evaluation/Data for Impact (D4I), University of North Carolina at Chapel Hill, USA, and the United States Agency for International Development (USAID)/Bangladesh determined that this study meets the definition of non-human subject research and exempted it from IRB oversight. Participants (Aged 15-19) provided verbal informed consent to participate in the survey."

2. Please provide separate figure files in .tif or .eps format.
   For more information about figure files please see our guidelines:
   https://journals.plos.org/globalpublichealth/s/figures
   https://journals.plos.org/globalpublichealth/s/figuresloc-file-requirements

   Ans: Thank you so much for your suggestion base on journal guidelines. I attached separate figure files in .tif format

3. Some material included in your submission may be copyrighted. According to PLOS's copyright policy, authors who use figures or other material (e.g., graphics, clipart, maps) from another author or copyright holder must demonstrate or obtain permission to publish this material under the Creative Commons Attribution 4.0 International (CC BY 4.0) License used by PLOS journals. Please closely review the details of PLOS's copyright requirements here: PLOS Licenses and Copyright. If you need to request permissions from a copyright holder, you may use PLOS's Copyright Content Permission form.
   Please respond directly to this email or email the journal office and provide any known details concerning your material's license terms and permissions required for reuse, even if you have not yet obtained copyright permissions or are unsure of your material's copyright compatibility.
   Potential Copyright Issues:
   S2 Figure: please (a) provide a direct link to the base layer of the map (i.e., the country or region border shape) and ensure this is also included in the figure legend; and (b) provide a link to the terms of use / license information for the base layer image or shapefile. We cannot publish proprietary or copyrighted maps (e.g. Google Maps, Mapquest) and the terms of use for your map base layer must be compatible with our CC-BY 4.0 license.
   Note: if you created the map in a software program like R or ArcGIS, please locate and indicate the source of the basemap shapefile onto which data has been plotted.
   If your map was obtained from a copyrighted source please amend the figure so that the base map used is from an openly available source. Alternatively, please provide explicit written permission from the copyright holder granting you the right to publish the material under our CC-BY 4.0 license.
   Please note that the following CC BY licenses are compatible with PLOS license: CC BY 4.0, CC BY 2.0 and CC BY 3.0, meanwhile such licenses as CC BY-ND 3.0 and

others are not compatible due to additional restrictions.
If you are unsure whether you can use a map or not, please do reach out and we will be able to help you. The following websites are good examples of where you can source open access or public domain maps: * U.S. Geological Survey (USGS) - All maps are in the public domain. (http://www.usgs.gov) * PlaniGlobe - All maps are published under a Creative Commons license so please cite "PlaniGlobe, http://www.planiglobe.com, CC BY 2.0" in the image credit after the caption. (http://www.planiglobe.com/?lang=enl) * Natural Earth - All maps are public domain. (http://www.naturalearthdata.com/about/terms-of-use/)

Ans: We are grateful for your insightful suggestion. The base layer used for Figure S2 was obtained from an open-source dataset (https://data.humdata.org/dataset/cod-ab-bgd) under CC-BY 4.0 license, and all subsequent spatial analyses were performed using the R programming environment version 4.5.1

Reviewer 1

1. The Introduction section and literature review should be separate. Mainly literature is discussed in Introduction section. It should be separated and grouped logically. Like few studies are discussed first from South Asia, then moved to UK studies and then back to China. Either group the studies geographically to discuss them or group the studies according to age or gender. For reference look at the paper "Prevalence of cyberbullying victimization among Pakistani Youth, by Sumera Saleem * , Naurin Farooq Khan, Saad Zafar". The way literature is grouped and discussed in this paper is more comprehensive and logical.

   Ans: We thank the reviewer for this valuable observation. We agree that the manuscript in its current form does not necessarily do justice to the value addition of the manuscript to the current body of academic knowledge. Indeed, this study's introduction and literature review updated base on more specific group such as global adolescents mental health, the South Asian context, Bangladesh context, bullying and mental wellbeing, physical bulling and mental wellbeing, verbal bulling and mental wellbeing, and cyberbullying and mental wellbeing.

   - **To improve the clarity of its introduction and literature review, we have incorporated a detailed on it. The amendment (page 2-6, lines 26-141) states:**

     "According to the World Health Organization (WHO), adolescence is a critical developmental stage that affects individuals

between the ages of 10 and 19, characterized by significant emotional, cognitive, and social changes. It is essential to keep an eye on this group's mental wellbeing for the sake of social advancement and public health.

...

These socio-demographic disparities underscore the urgent need for targeted interventions and highlight the importance of considering school, age, wealth, gender, and marital status when assessing the relationship between bullying and mental health."

- The justification of conducting this research should be stronger, as the highlighted following red section is needed. Why association between forms of bullying and depression need to be done in Bangladesh.
  Previously several studies have measured depression in the context of bullying[10,18,19], but no research in Bangladesh has specifically examined the association between physical bullying, verbal bullying, and cyberbullying and depression among adolescents.

  Ans: We thank the reviewer for pointing out the issue. We agree that the manuscript in its current form does not necessarily explain the justification. In response, we have incorporated an elaborate discussion (page 6-7, lines 143-164) on justification that states:

  "Several studies in Bangladesh have explored the relationship between bullying, violence, and adolescent mental health separately. In a study it was examined that how experiences of sexual harassment contribute to depression among adolescent girls in rural Bangladesh

  ...

  In this study, the hierarchical structure of the data has been incorporated through the development of a multilevel model to account for both cluster- and district-level variation. Thus, the study aims to assess the influence of different forms of bullying on adolescent mental well-being in Bangladesh while considering socio-demographic, school, and contextual factors. Unlike prior research, it employs multistage modeling techniques to explore these relationships, an approach not previously utilized in this context."

- The research methods section is poorly structured. It should be well structured. See the following papers for reference https://doi.org/10.1057/s41284-025-00470-8

Ans: We thank the reviewer for pointing out the issue. We agree that the manuscript in its current form does not necessarily explain the linear mixed model. In response, we have incorporated an elaborate discussion (page 9-10, lines 235-255) in methods section that states:

> ***"The linear mixed model, also known as a multilevel model, is an extension of the linear model where the random effects of hierarchical levels (such as villages and regions where an individual belongs to) are considered in addition to the fixed effects of factors and covariates, which were assumed to have a linear relationship with the dependent variable [56]. In this study, we employed a three-level mixed-effects regression model.***
>
> ...
>
> ***The fitted models can be used to estimate mean and distribution functions of a mental well-being at different hierarchies (for example, PSU and region in this study) [52]. The multilevel models were developed using proc mixed in SAS [57]."***

- I have concern about hypothesis formation.
  a. They should not be in Introduction section, make separate sections of Present Study and include Hypothesis there.
  b. Why you are not considering the "married male " the selection of three groups needs some justification. And exclusion of mentioned group also needs reason.
  c. Moreover, try to formulate good hypotheses. See in reference studies.
  d. Where you have proven your hypothesis true or false. I am unable to find in the study that H1 is proven true or false.
  e. Split your hypothesis 2 in three sub-Hypothesis with each group separately.
  f. You are also proving depression and bullying with reference to socio economic status, you can make your hypothesis surrounding those factors as well.

  Ans: I appreciate the concern about the clarity of hypothesis formation.
  - **a. We appreciate your suggestion. In line with your valuable suggestion, we have incorporated an additional subsection into the present study and made necessary amendments (lines 143-154; page 06-07) in the manuscript that states:**

    > ***"Several studies in Bangladesh have explored the relationship between bullying, violence, and adolescent mental health separately. In a study it was examined that how experiences of sexual harassment contribute to depression among adolescent girls in rural Bangladesh [28,31]. In other study focused on the prevalence of cyberbullying and its strong as-***

sociation with depressive disorders among adolescents[43] . "

...

Research Hypotheses:
Hypothesis 1: Forms of bullying are associated with adolescent mental health.
Hypothesis 2: Physical, verbal, and cyberbullying are associated with mental health among ever-married female adolescents.
Hypothesis 3: Physical, verbal, and cyberbullying are associated with mental health among unmarried female adolescents.
Hypothesis 4: Physical, verbal, and cyberbullying are associated with mental health among unmarried male adolescents.
Hypothesis 5: Sociodemographic factors are associated with mental health. "

- **b. We would like to thank the reviewer for highlighting the issue regarding the exclusion of married males from our analysis. As stated in the data source section, we utilized secondary data from the Bangladesh Adolescent Health and Wellbeing Survey (BAHWS), which did not include information on married males. We acknowledge that inclusion of such data could have allowed for a more comprehensive comparative analysis. Accordingly, this has been noted as a limitation of our study. In response, we have incorporated amendments (lines 429-34; page 17-18) to clarify the stance of the study.**

- **c. Thank you for the valuable suggestion and reference regarding the hypothesis. We appreciate your suggestion regarding the clarity of the study to formulate a hypothesis that better guides the direction of the study.**

- **d. The discussion section was thoroughly revised to align with the stated hypotheses and to incorporate pertinent findings from previous studies.**

- **e. We thank you for your valuable suggestion. Based on your feedback, we have divided the original Hypothesis 2 into three separate hypotheses (2 to 4), which allowed us to obtain more detailed and insightful findings.**

- **f. We appreciate the reviewer's thoughtful recommendation. As a result, a new hypothesis 5 has been formulated, incorporating socio-economic status to strengthen the analytical framework.**

- Discussion and conclusion will be around proving your hypothesis. I would suggest the discussion should be strongly with reference to Bangladesh cultures, issues policies in comparison to relevant studies.

  Ans: We thank the reviewer for this well-thought-out suggestion. While our manuscript provides detailed quantitative findings, we recognize the need for greater clarity about their hypothesis. In response, we have improved the narrative and hypothesis around the implications of key statistical results.
  We have substantially revised the entire Discussion and Conclusions section to address the concern. The major amendment (Page 14-18, Lines 333–451) includes a detailed analysis of the implications of the major findings in line with the hypothesis within the broader academic literature. We have also expanded the discussion to highlight the relevance of these results for public health concerns and the development of early warning systems.

General: We thank all reviewers for their constructive suggestions and comments to improve the quality of the paper.

Yours sincerely,

Ruhul Amin

Staff Editor

PLOS Global Public Health
