# Supplementary material for: Exploring the association between bullying and depression symptoms among adolescents in Bangladesh: Findings from nationally representative survey
Source: PLOS Ment Health. 2026 Oct 28;3(10):e0000722. doi: 10.1371/journal.pmen.0000722 (PMC13716191; doi:10.1371/journal.pmen.0000722)
Supplement: Supplementary file 2 [file pmen.0000722.s008.pdf]

Ruhul Amin
Research Associate
Bangladesh Institute of Governance and Management
Dhaka-1207, Bangladesh
E-mail: ruhul.aminbigm.edu.bd

July 5, 2026

Academic Editor
Plos Mental Health

Dear Dr. Helena R. Slobodskaya,

Please find the revised version of the manuscript (Submission ID: PMEN-D-26-00157) entitled: **"Exploring the Association Between Bullying and Depression Symptoms Among Adolescents in Bangladesh: Findings from Nationally Representative Survey"**, and consider it for publication in the journal **Plos Mental Health**.

We have revised the manuscript according to the reviewer's comments and suggestions.

Authors' Note: The major corrections and new addendum are highlighted in blue color texts for Journal Requirements, magenta color texts for Editorial Board Member(EBM), red color texts for Reviewer 1 and teal color texts for Reviewer 2 in the revised manuscript. Additional corrections are highlighted in **a different color** following the journal template.

Legend (text colors for revision and corrections)

- Journal Requirements
- EBM
- Reviewer 1
- Reviewer 2

Journal Requirements

1. Please note that PLOS Mental Health has specific guidelines on code sharing for submissions in which author-generated code underpins the findings in the manuscript. In these cases, we expect all author-generated code to be made available without restrictions upon publication of the work. Please review our guidelines at https://journals.plos.org/mentalhealth/s/materials-and-software-sharing#loc-sharing-code

and ensure that your code is shared in a way that follows best practice and facilitates reproducibility and reuse.

Ans: Thank you for highlighting the PLOS Mental Health code-sharing policy. In accordance with the journal's guidelines, we have made all author-generated analysis code publicly available through a GitHub repository to facilitate transparency, reproducibility, and reuse. The GitHub repository link has been added to the Data Availability Statement (https://github.com/ruhulsta/Adolescent-bullying-and-depression-in-Bangladesh**) in the revised manuscript. The repository includes the complete SAS and R scripts used for data management, statistical analyses, and figure generation. We trust that this revision fully addresses the journal's code-sharing requirements.**

2. Thank you for uploading your study's underlying data set. Unfortunately, the repository you have noted in your Data Availability statement does not qualify as an acceptable data repository according to PLOS's standards. At this time, please upload the minimal data set necessary to replicate your study's findings to a stable, public repository (such as figshare or Dryad) and provide us with the relevant URLs, DOIs, or accession numbers that may be used to access these data. For a list of recommended repositories and additional information on PLOS standards for data deposition, please see https://journals.plos.org/plosone/s/recommended-repositories.

   Ans: Thank you for your comment. To facilitate reproducibility, we have made available the minimal anonymized dataset required to reproduce the analyses and main findings together with the complete analysis code in a public GitHub repository (https://github.com/ruhulsta/Adolescent-bullying-and-depres **. The dataset includes the variables used in the statistical analyses while excluding unnecessary information to protect participant confidentiality. The revised Data Availability Statement has been updated accordingly. If required by the journal, we are happy to deposit the same replication dataset in a PLOS-recommended public data repository.**

3. Some material included in your submission may be copyrighted. According to PLOS's copyright policy, authors who use figures or other material (e.g., graphics, clipart, maps) from another author or copyright holder must demonstrate or obtain permission to publish this material under the Creative Commons Attribution 4.0 International (CC BY 4.0) License used by PLOS journals. Please closely review the details of PLOS's copyright requirements here: PLOS Licenses and Copyright. If you need to request permissions from a copyright holder, you may use PLOS's Copyright Content Permission form. Please respond directly to this email or email the journal office and provide any known details concerning your material's license terms and permissions

required for reuse, even if you have not yet obtained copyright permissions or are unsure of your material's copyright compatibility. Potential Copyright Issues: a. S2 Fig: please (a) provide a direct link to the base layer of the map (i.e., the country or region border shape) and ensure this is also included in the figure legend; and (b) provide a link to the terms of use / license information for the base layer image or shapefile. We cannot publish proprietary or copyrighted maps (e.g. Google Maps, Mapquest) and the terms of use for your map base layer must be compatible with our CC-BY 4.0 license. Note: if you created the map in a software program like R or ArcGIS, please locate and indicate the source of the basemap shapefile onto which data has been plotted. If your map was obtained from a copyrighted source please amend the figure so that the base map used is from an openly available source. Alternatively, please provide explicit written permission from the copyright holder granting you the right to publish the material under our CC-BY 4.0 license. Please note that the following CC BY licenses are compatible with PLOS license: CC BY 4.0, CC BY 2.0 and CC BY 3.0, meanwhile such licenses as CC BY-ND 3.0 and others are not compatible due to additional restrictions. If you are unsure whether you can use a map or not, please do reach out and we will be able to help you. The following websites are good examples of where you can source open access or public domain maps: * U.S. Geological Survey (USGS) - All maps are in the public domain. (http://www.usgs.gov) * PlaniGlobe - All maps are published under a Creative Commons license so please cite "PlaniGlobe, http://www.planiglobe.com, CC BY 2.0" in the image credit after the caption. (http://www.planiglobe.com/?lang=enl) * Natural Earth - All maps are public domain. (http://www.naturalearthdata.com/about/terms-of-use/)

Ans: Thank you for bringing this to our attention. The map used in S2 Fig was created by the authors in R using an openly available Bangladesh administrative boundary shapefile obtained from humanitarian data exchange (hdx). We will revise the figure legend to include the direct URL to the shapefile, its license/terms of use, and the appropriate attribution to ensure compliance with the CC BY 4.0 requirements. If necessary, we will replace the basemap with a fully compatible open-access source.

EBM

1. First, the division of the sample into subsamples based on gender and marital status (married females, unmarried females and unmarried males) seems rather unsubstantiated and serves as the manuscript main weakness. I would suggest to include adolescent gender and marital status as explanatory variables in addition to age, residence, wealth and school/college attendance. It would be helpful to see the gender and marital status distribution in Table 1 along with gender and marital status differences in Table 2. Figures 1 and 2, by contrast, do not seem to be informative and should be removed.

Ans: hank you for this thoughtful and constructive comment. We have

substantially revised the manuscript to address each of these concerns.
First, as suggested, gender and marital status were included as explanatory variables in the primary three-level linear mixed-effects model, together with age, residence, household wealth, school attendance, division, and internet device use. To formally assess whether the associations between bullying victimization and depressive symptoms differed by gender, we additionally incorporated interaction terms between gender and each bullying type (physical, verbal, and cyberbullying). These analyses demonstrated significant gender interactions for physical and verbal bullying, whereas the interaction for cyberbullying was not statistically significant.
Second, we expanded the descriptive analyses by adding the distributions of gender, marital status, and gender–marital status groups to Table 1, and by presenting the corresponding differences in mean depressive symptom scores in Table 2, as recommended.
Third, we clarified the methodological rationale for the subgroup analyses in the Methods section. The subgroup analyses were conducted because the Bangladesh Adolescent Health and Wellbeing Survey includes three adolescent populations—ever-married females, unmarried females, and unmarried males—while married adolescent males were not represented in the survey. Accordingly, the subgroup analyses were intended as exploratory analyses to complement the primary model and to examine whether the observed associations varied across the available adolescent groups. Throughout the revised manuscript, we have interpreted these subgroup findings cautiously and in conjunction with the formal interaction analyses.
Finally, following the reviewer's recommendation, we removed the previous descriptive figure (Figure 1). However, we respectfully retained the forest plot (now Figure 1), as it summarizes the adjusted associations between different forms of bullying and depressive symptoms across the three predefined adolescent groups. This figure directly addresses the study objective and Hypothesis 2 by facilitating comparison of subgroup-specific associations and complements the interaction analyses presented in the main text. These revisions have been incorporated throughout the Introduction, Methods, Results, Discussion

2. I agree with Reviewer 2 that it is important to consider multiple types of bullying and to examine the effects of bullying in more detail. It might be helpful to create a composite measure of bullying and to compare the effects of specific types of bullying and the composite measure.

   Ans: Thank you for this valuable suggestion. We fully agree that examining both individual forms of bullying and cumulative bullying exposure provides a more comprehensive understanding of their associations with

depressive symptoms.

In response, we created a composite bullying variable based on the number of bullying types experienced by each adolescent and categorized participants into four groups: no bullying, one type, two types, and three types of bullying. We then fitted an additional three-level linear mixed-effects model using this composite measure while adjusting for the same covariates included in the primary analysis.

The results of this additional analysis are presented in the newly added Table 5. We observed a clear dose–response relationship, whereby adolescents exposed to one, two, and three types of bullying had progressively higher depressive symptom scores compared with those reporting no bullying exposure. This finding complements the analyses of individual bullying types (physical, verbal, and cyberbullying) presented in the primary model and provides additional evidence that cumulative bullying victimization is associated with a greater burden of depressive symptoms.

The Methods, Results, Discussion, and Conclusion sections have been revised accordingly to incorporate and interpret these additional findings.

3. It is unclear from the Statistical Analysis subsection and the Results whether the coefficients in Table 3 present results from a series of linear mixed models, each with one predictor variable, or results from one linear mixed model with multiple predictors.

   Ans: Thank you for this important comment. We agree that the original description was not sufficiently clear. We have revised the Statistical Analysis subsection to explicitly state that the reported regression coefficients were estimated from a single multivariable three-level linear mixed-effects model that simultaneously included all bullying variables, interaction terms, and covariates. Specifically, the model adjusted for age, residence, household wealth, school attendance, division, marital status, gender, internet device use, and the bullying variables, while accounting for clustering at the PSU and district levels.

   We also revised the Results section to clarify that the coefficients presented in Table 4 were obtained from this single adjusted multivariable model rather than from separate models for each predictor. These revisions improve the transparency and interpretation of the regression analyses.

4. I strongly recommend to examine all possible explanatory variables in one regression model including: first, main effects of bullying, socioeconomic and demographic variables; and, second, bivariate interactions of bullying, socioeconomic and demographic variables. I agree with Reviewer 1 that interaction tests are crucial for understanding how the association of bullying with depressive symptoms varies across socio-demographic groups.

   Ans: Thank you for this valuable suggestion. We agree that interaction analyses are important for understanding whether the associations between bullying victimization and depressive symptoms differ across sociodemo-

graphic groups.
In response, we revised the primary analysis by fitting a single multivariable three-level linear mixed-effects model that simultaneously included all bullying variables (physical bullying, verbal bullying, and cyberbullying), the prespecified sociodemographic covariates (age, residence, household wealth, school attendance, division, marital status, gender, and internet device use), and interaction terms.
Because one of the principal objectives of this study was to examine differences in bullying–depression associations across adolescent sex and marital status groups, we specifically evaluated gender × bullying interaction terms for physical bullying, verbal bullying, and cyberbullying. These analyses demonstrated significant interactions for physical and verbal bullying, whereas the interaction for cyberbullying was not statistically significant. The subgroup analyses were interpreted in conjunction with these formal interaction tests.The Methods, Results, Discussion, and Table 4 have been revised accordingly.

5. Finally, I would suggest to present comparisons between 2- and 3-level mixed models in the preliminary analysis subsection. Although this might be valuable information, the focus of this study is on the relations of bullying and depressive symptoms in adolescents from different socio-demographic groups.
Ans: Thank you for this helpful suggestion. We agree that the comparison between the two-level and three-level mixed-effects models is part of the model-building process rather than the primary findings of the study.
Accordingly, we have moved the comparison of the two-level and three-level mixed-effects models (including the variance components, model fit statistics, intraclass correlation coefficients, and likelihood ratio test) from the main Results section to the Preliminary analysis subsection. This reorganization improves the logical flow of the manuscript by presenting the model selection process before the primary multivariable analyses and allows the main Results section to focus on the associations between bullying victimization and depressive symptoms across different sociodemographic groups. The manuscript has been revised accordingly.

Reviewer 1

1. The introduction requires substantial restructuring and editing. At present, it is too broad, repetitive, and conceptually inconsistent. The manuscript moves back and forth between "mental health," "mental well-being," "depression," and even broader psychological outcomes, but the actual outcome analyzed is a PHQ-9 depression score. The introduction should therefore be rewritten around a much more focused question: bullying and depressive symptoms among Bangladeshi adolescents. The long global overview is not well integrated into the study rationale, and the Bangladesh-specific

gap appears too late.

Ans: Thank you for this insightful and constructive comment. We agree that including gender and marital status as explanatory variables in the multivariable analysis provides a more rigorous assessment of their independent associations with adolescent depressive symptoms and strengthens the interpretation of subgroup differences.
Accordingly, we substantially revised the analytical framework. Gender and marital status were included as explanatory variables in the primary three-level linear mixed-effects model together with age, place of residence, household wealth, school/college attendance, division, internet device use, and other relevant covariates. In addition, we incorporated interaction terms between gender and each bullying type (physical bullying, verbal bullying, and cyberbullying) to formally evaluate whether the associations between bullying victimization and depressive symptoms differed by gender. The subgroup analyses were retained as complementary exploratory analyses and are now interpreted alongside the formal interaction tests.
To improve the descriptive presentation of the study population, we updated Table 1 to include the distributions of gender, marital status, and gender–marital status groups. Table 2 was also revised to present differences in depressive symptoms across these groups, providing greater context for interpreting the multivariable regression findings.
Following this recommendation, we also revised the conceptual framing of the manuscript. Specifically, the title, research objectives, and study hypotheses were updated to align with the revised analytical framework and to clearly identify depressive symptoms as the primary outcome. Accordingly, the manuscript title has been changed to
"Exploring the Association Between Bullying and Depressive Symptoms Among Bangladeshi Adolescents: Findings from a Nationally Representative Survey",
which more accurately reflects the study objective, outcome measure, and scope of the analysis.
Regarding the figures, we removed the previous Figure 1 as suggested. However, we respectfully retained the forest plot (Figure 2) because it provides a concise visual summary of the adjusted associations between different forms of bullying and depressive symptoms across the three predefined adolescent groups. This figure directly addresses the revised study objective and Hypothesis 2, complements the interaction analyses, and facilitates interpretation of the subgroup findings. The figure has also been improved to enhance clarity and readability.
These revisions have been incorporated throughout the Title, Introduction, Research Objectives and Hypotheses, Methods (Page 7, Lines 182–289), Table 1 (Page 11), Table 2 (Page 13-14), and Figure 1 of the revised

manuscript.

2. There are also major writing problems. Some passages are redundant or awkwardly phrased, and several sentences require editing for clarity and grammar. A clear example is the repeated England school-survey sentence in the physical bullying section, where essentially the same point is stated twice consecutively. The subsection "In Present Study" is also poorly written and reads as a patchwork summary rather than a coherent rationale.

   Ans: Thank you for this valuable observation. We carefully reviewed the entire manuscript to improve its overall clarity, coherence, and readability. In response to your comment, we thoroughly revised the Introduction and other relevant sections to eliminate redundant statements, correct grammatical issues, and improve sentence structure and flow.
   Specifically, the repeated statement referring to the England school survey in the physical bullying section has been removed, and the surrounding text has been rewritten to present the evidence more concisely and logically. In addition, we revised several awkwardly phrased sentences throughout the manuscript to enhance clarity and readability.
   Furthermore, the subsection previously titled "In Present Study" has been substantially rewritten and reorganized. Rather than presenting a collection of disconnected statements, the revised section now provides a coherent rationale that clearly identifies the existing knowledge gap, explains the importance of examining the association between bullying and adolescent mental well-being in the Bangladeshi context, and explicitly states the study objectives and hypotheses. The revised Introduction now follows a more logical progression from the global evidence on bullying and mental health to the specific context and motivation for the present study.
   Overall, these revisions have substantially improved the manuscript's organization, clarity, and narrative flow. The relevant changes have been incorporated throughout the Introduction and other sections of the revised manuscript (Pages 04–05, Lines 96–130).

3. The novelty and gap statements are overstated. The authors claim that "no research in Bangladesh" has examined these associations, yet the introduction itself cites Bangladesh studies linking cyberbullying, violence, and depression. A more defensible claim would be that this study integrates multiple bullying forms in one national analysis and examines available subgroups within the BAHWS dataset. The current framing overstates what is new.

   Ans:Thank you for this thoughtful comment. We agree that the original wording overstated the novelty of the study. In the revised manuscript,

we have modified the introduction to acknowledge the existing Bangladeshi literature examining bullying, violence, cyberbullying, and mental health outcomes among adolescents.
Rather than suggesting a complete absence of prior research, we now emphasize the specific contribution of the present study. Specifically, the revised text highlights that previous studies have generally focused on individual forms of bullying or have not fully accounted for the hierarchical structure of the data. In contrast, our study simultaneously examines physical bullying, verbal bullying, and cyberbullying within a nationally representative sample and applies a multilevel modeling framework to account for clustering at the cluster and district levels. This approach enables the assessment of both individual- and contextual-level influences on adolescent mental well-being.
Accordingly, we have revised the gap statement and study rationale to provide a more balanced and accurate description of the study's contribution. These revisions have been incorporated in the Introduction (Page 05, Lines 96–117).

4. The hypotheses also need revision. They are framed around "mental health," although the modeled outcome is depression. They are also largely descriptive and are not tightly linked to a conceptual framework. I would recommend either simplifying the aims or reformulating the hypotheses so they directly match the measured outcome and planned subgroup comparisons.

   Ans: Thank you for this constructive suggestion. We agree that the original hypotheses were not sufficiently aligned with the primary outcome of the study. Accordingly, we have revised both the study aim and hypotheses to correspond directly to the measured outcome—depressive symptoms assessed using the PHQ-9 depression score.
   Specifically, we replaced the broad term "mental health" with "depressive symptoms" throughout the hypotheses and reformulated them to reflect the study's analytical framework. These revisions ensure consistency between the study objectives, hypotheses, statistical analyses, and interpretation of the findings.
   The revised aim and hypotheses have been incorporated into the Introduction (Page 05, Lines 118–30).

5. The most important conceptual problem in the methods is the outcome definition. The manuscript repeatedly calls the outcome "mental wellbeing," but the outcome is a summed modified PHQ-9 score. That is a measure of depressive symptom severity, not general mental well-being. This needs to be corrected throughout the manuscript. In addition, the authors state that they used a "modified" PHQ-9, but they do not

explain what was modified, whether the adapted version was validated in this setting, or what reliability was observed in the analytic sample.

Ans: Thank you for this important comment. We agree that the outcome should be described more precisely. Accordingly, we have revised the manuscript throughout to replace the broad terms "mental wellbeing" and "mental health" with "depressive symptoms" or "PHQ-9 depression score," as appropriate, ensuring consistency with the outcome analyzed in this study.
We have also substantially expanded the description of the outcome measure in the Methods section. Specifically, we now explain the adolescent-modified PHQ-9, describe its scoring procedure, clarify that the depression score was calculated by summing the nine core PHQ-9 items, and report the score range and interpretation.
Furthermore, we have added information on the psychometric properties of the Bangla-adapted PHQ-9, including previous validation studies conducted in Bangladesh. In addition, we calculated the internal consistency of the PHQ-9 in our analytical sample and now report the observed Cronbach's alpha ($\alpha = 0.837$), demonstrating good reliability in the present study.
These revisions provide a clearer description of the outcome measure and ensure that the terminology and interpretation are fully aligned with the depressive symptom outcome analyzed in this study (Page 06-07, Lines 162–176).

6. The sample selection also requires much clearer reporting. The survey initially identified 18,249 adolescents, but only 9,116 were included because they had "plausible information" for calculating the outcome. Excluding roughly half of the identified sample is not a minor issue. The authors need to explain exactly why so many participants were excluded, provide a missing-data description, and discuss the possibility of selection bias. A comparison between included and excluded cases would strengthen the paper substantially.

Ans: Thank you for this important comment. We agree that the sample selection process required further clarification. Accordingly, we have revised the Data Source section to explicitly describe the derivation of the analytical sample. Specifically, we now clarify that the study outcome was based on the PHQ-9 depression score, and only adolescents with complete and valid responses to all PHQ-9 items were eligible for inclusion. Participants with incomplete PHQ-9 responses could not have a valid depression score calculated and were therefore excluded from the analytical sample.
We have also added a description of the handling of missing data, noting that missing values in explanatory variables were addressed using complete-

case analysis during model estimation, whereas the primary reduction in sample size resulted from missing outcome data. In addition, we have expanded the Discussion section to acknowledge that excluding participants with missing outcome information may have introduced selection bias if the missingness was not completely random, and we have identified this as a limitation of the study.

Following your suggestion, we also compared the characteristics of included and excluded participants using variables available for both groups. The comparison is presented in Supplementary Table S1. These revisions have been incorporated into the revised manuscript (Page 11, Lines 277-299); Supplementary Table S1; Discussion (Page 21, Lines 524–30).

7. There is also an important mismatch in time frames: bullying exposures were assessed over the previous 12 months, whereas depressive symptoms were assessed over the previous 2 weeks. This does not invalidate the analysis, but it is a key design limitation and should be acknowledged explicitly, because it weakens temporal interpretation.

   Ans: Thank you for this insightful comment. We agree that the differing assessment periods for bullying victimization and depressive symptoms represent an important limitation of the study. In the revised manuscript, we have explicitly acknowledged this issue in the Discussion.

   Specifically, we now clarify that bullying victimization was assessed over the preceding 12 months, whereas depressive symptoms were measured using the PHQ-9 over the preceding two weeks. Consequently, although the study identifies significant associations between bullying victimization and depressive symptoms, the differing recall periods limit temporal interpretation and preclude causal inference. We therefore emphasize that the findings should be interpreted as associations rather than evidence of a temporal or causal relationship. We have also highlighted the need for future longitudinal studies with aligned assessment periods to better establish the temporal relationship between bullying victimization and depression.

   These revisions have been incorporated into the Limitations section of the Discussion (Page 21, Lines 527–33).

8. The statistical analysis section needs clarification. The authors state that sample weights were applied and that a multilevel linear mixed model was used, but it is not clearly explained how the complex survey design and weighting were incorporated into the multilevel analysis. For nationally representative survey data, this point is methodologically important. In addition, the choice of a linear mixed model for a bounded symptom score should be justified more explicitly, and model diagnostics or sensitivity analyses would be helpful.

Ans: Thank you for this valuable methodological comment. We have substantially revised the Statistical Analysis section to clarify the specification of the multilevel model, the incorporation of the complex survey design, and the rationale for model selection.
Specifically, we now explicitly describe that the Bangladesh Adolescent Health and Wellbeing Survey employed a multistage sampling design in which adolescents were nested within primary sampling units (PSUs) and districts. To account for this hierarchical structure, we fitted a three-level random-intercept linear mixed-effects model with adolescents (level 1) nested within PSUs (level 2), and PSUs nested within districts (level 3). Individual sampling weights provided by the survey were incorporated during model estimation using the WEIGHT statement in SAS PROC MIXED to account for unequal probabilities of selection, while district- and PSU-level random intercepts accounted for the clustering introduced by the sampling design.
We also expanded the description of the statistical model by providing the mathematical formulation of the three-level mixed-effects model and clarifying the assumptions regarding the random effects and variance components.
Furthermore, we have strengthened the justification for using a linear mixed-effects model. The PHQ-9 depression score was analyzed as a continuous measure of depressive symptom severity, and linear mixed-effects models are widely used for continuous psychometric outcomes while appropriately accommodating hierarchical data structures and correlated observations.
Finally, we have added a description of the model diagnostic procedures. Model assumptions were evaluated by examining residual and quantile–quantile (Q–Q) plots to assess the normality and homoscedasticity of residuals. These diagnostic assessments indicated no substantial violations of the model assumptions. The relevant revisions have been incorporated into the Statistical Analysis section (Page 09-10, Lines 243–75).

9. Finally, the subgroup structure needs a clearer methodological rationale. The manuscript analyzes ever-married females, unmarried females, and unmarried males, while acknowledging that married adolescent boys were unavailable in the dataset. That limitation is noted later, but the methods should explain the subgroup strategy more transparently at the outset.

   Ans: Thank you for this valuable suggestion. We agree that the rationale for the subgroup analyses should be presented more clearly in the Methods section. Accordingly, we have revised the manuscript to explain that the subgroup analyses were conducted to explore potential differences in the associations between bullying experiences and depressive symptoms according to sex and marital status, based on the demographic composition

of the Bangladesh Adolescent Health and Wellbeing Survey (BAHWS). We now explicitly state that the BAHWS did not include married adolescent boys because marriage among adolescent males is uncommon in Bangladesh, where the legal minimum age of marriage is 21 years for males. Consequently, the available data permitted subgroup analyses only among ever-married females, unmarried females, and unmarried males. In addition, we have acknowledged this as a study limitation in the Discussion, noting that the subgroup findings cannot be generalized to married adolescent males or other adolescent populations not represented in the survey. These revisions have been incorporated into the Methods (Page 09, Lines 233–41) and the Limitations section of the Discussion (Page 21, Lines 518–22).

10. The results section contains reporting problems that need correction before the findings can be evaluated with confidence. Most notably, the text describing Table 3 states that physical bullying, verbal bullying, and cyberbullying were associated with depression by "2.11, 2.11, and 2.11," whereas Table 3 shows coefficients of 1.12, 1.30, and 2.11. This is a direct inconsistency between the text and the table.
Ans: Thank you for carefully identifying this inconsistency. We agree that the discrepancy resulted from a typographical error in the Results section. The text has been corrected to ensure complete consistency with the estimates presented in Table 3. Specifically, the coefficients for physical bullying, verbal bullying, and cyberbullying have been revised. We have also carefully reviewed the Results section to verify the consistency of all numerical values reported in the text with the corresponding tables and figures.

11. There are also typographical or data-presentation errors in the descriptive tables. For example, in Table 1 the weighted percentage for age 17 appears as "2074," which is presumably 20.74. Errors of this kind undermine confidence in the rigor of the results section and should be carefully corrected throughout.

    Ans: Thank you for identifying this typographical error. We agree that accurate data presentation is essential for ensuring the clarity and credibility of the results. The weighted percentage for age 17 in Table 1 has been corrected from "2074" to "20.74". In addition, we have carefully reviewed all tables, figures, and the corresponding text throughout the manuscript to identify and correct any similar typographical or formatting errors. These revisions ensure that the reported values are presented accurately and consistently throughout the manuscript. The correction has been incorporated into Table 1 of the revised manuscript.

12. The subgroup results are potentially interesting, but the authors overinterpret between-group differences. They describe some bullying forms as having larger effects in certain

subgroups, yet I do not see formal interaction tests showing that these differences between ever-married females, unmarried females, and unmarried males are statistically different from one another. Without interaction analyses, these comparisons should be described more cautiously.

Ans: Thank you for this insightful comment. We agree that subgroup-specific regression coefficients alone do not provide formal evidence that the associations differ between groups. In response, we revised the analytical approach by including interaction terms between gender and each bullying exposure in the multilevel model, while simultaneously adjusting for gender, marital status, and other covariates. The interaction analyses indicated statistically significant interactions for physical bullying (β= 1.49, $p < 0.001$) and verbal bullying ($\beta = 0.86$, $p < 0.001$), whereas the interaction between cyberbullying and gender was not statistically significant (β= 0.92, $p = 0.064$).
We did not additionally model interactions with marital status because the subgroup classification is inherently defined by the combination of gender and marital status. Simultaneously fitting subgroup-specific interactions together with their defining variables would introduce substantial redundancy and potential multicollinearity, thereby complicating model interpretation. Furthermore, we have revised the Results and Discussion sections to avoid overinterpreting subgroup differences. The subgroup analyses are now presented as exploratory analyses describing potential heterogeneity in the estimated associations, whereas formal inference regarding between-group differences is based on the interaction analyses. The corresponding interpretations throughout the manuscript have been revised accordingly (Pages 14-15, Lines 341 - 68).

13. The multilevel model results also need to be interpreted more carefully. The 3-level model shows somewhat better fit than the 2-level model, but the improvement appears modest, and the ICC changes only from 0.030 to 0.033. This supports the use of a multilevel approach, but not a strong claim that the model meaningfully transformed the substantive conclusions.

    Ans: Thank you for this thoughtful comment. We agree that the improvement in model fit should be interpreted cautiously and should not be overstated. In the revised manuscript, we have carefully revised the Results and Discussion to provide a more balanced interpretation of the multilevel model comparison.
    To strengthen the model comparison, we now report additional model fit statistics, including the AIC, AICC, BIC, and -2 log likelihood values. We also performed a likelihood ratio test, which is the appropriate formal

method for comparing nested multilevel models. The results showed that the three level model provided a statistically significantly better fit than the two-level model ($\chi^2 = 16.7$, $p < 0.001$).
At the same time, we explicitly acknowledge that the increase in the intraclass correlation coefficient (ICC) from 3.19% to 3.30% was modest. Accordingly, we no longer imply that the three level model substantially changed the substantive conclusions. Instead, we emphasize that the three level model was retained because it more appropriately accommodated the hierarchical structure of the BAHWS data by accounting for variation at both the district and cluster levels while providing a statistically better fit to the data.
These revisions have been incorporated into the Results section page (14).

14. The discussion substantially overreaches the data. The authors frequently revert to "mental health" and "mental well-being" language, even though the outcome modeled is depression. They also refer to anxiety, suicidal ideation, and broader psychosocial consequences that were not directly analyzed in this study. The discussion should remain centered on what was actually measured: depressive symptoms.

    Ans: Thank you for this important observation. We agree that the Discussion should remain closely aligned with the outcome measured in this study. In response, we have carefully revised the manuscript to ensure that the terminology is consistent with the PHQ-9 depression score, which was the primary outcome of our analyses.
    Specifically, we have revised the manuscript title and thoroughly reviewed the Introduction, Methods, Results, Discussion, and Conclusion to replace broad terms such as "mental health" and "mental well-being" with more precise terminology referring to depressive symptoms or PHQ-9 depression scores, where appropriate. We have also removed or substantially revised statements referring to anxiety, suicidal ideation, and other broader psychosocial outcomes that were not directly measured in this study. The Discussion has been refocused on interpreting the observed associations between different forms of bullying and depressive symptoms without extending the conclusions beyond the available data.
    These revisions improve the consistency between the study objectives, outcome measure, statistical analyses, and interpretation of the findings. The relevant changes have been incorporated throughout the revised manuscript.

15. Several interpretations are not fully supported by the presented results. For example, the discussion states that there were significant associations with "gender," but gender is not presented as a direct covariate in the all-adolescent model shown in Table 3; rather, the paper presents separate subgroup analyses. Similarly, the discussion de-

scribes a “U-shaped” relationship between wealth and depression, but in the adjusted model the richest group is not significantly different from the poorest group. That interpretation should therefore be toned down.

Ans: Thank you for this thoughtful comment. We agree that the Discussion should closely reflect the adjusted model results. In response, we have revised both the Results and Discussion sections accordingly.
First, gender has now been included as a covariate in the overall multilevel model, and the corresponding results are presented in the revised Table 3. The Discussion has been updated to reflect the findings from the adjusted model rather than relying solely on the subgroup analyses.
Second, we have removed the description of a ”U-shaped” relationship between household wealth and depressive symptoms. The revised Discussion now more cautiously states that adolescents from poorer, middle, and richer households had significantly lower depression scores than those from the poorest households, whereas the difference for the richest wealth group was not statistically significant. We therefore acknowledge that the association between household wealth and depressive symptoms is complex and should not be interpreted as evidence of a U-shaped relationship.
These revisions ensure that the interpretation is fully consistent with the adjusted multivariable findings presented in the revised manuscript (Page 20, Lines 488–501).

16. The statement that cyberbullying was the “most damaging” form of bullying is also too strong for a cross-sectional analysis. The data support that cyberbullying had the strongest observed association with depressive symptoms in these models, but “most damaging” implies a causal and comparative claim that the design cannot establish. The same issue appears again in the conclusions.

Ans: Thank you for this important comment. We agree that the phrase ”most damaging” overstated the interpretation of our findings and could be interpreted as implying a causal relationship that cannot be established using a cross-sectional study design.
Accordingly, we have revised the Discussion and Conclusion to use more precise, non-causal language. Specifically, we replaced expressions such as ”most damaging” with ”strongest observed association with depressive symptoms” or equivalent wording throughout the manuscript. We have also revised the surrounding text to emphasize that the findings represent statistical associations rather than causal effects. These revisions ensure that the interpretation is fully consistent with the study design and the results of the multilevel analyses.
The relevant changes have been incorporated into the Discussion and Con-

clusion sections (Page 21, Lines 537–46).

17. The novelty claim is again overstated in the discussion. The manuscript presents the multilevel modeling approach as a unique contribution, but the model fit statistics suggest only a marginal gain. The paper may have incremental value as a national Bangladesh analysis, but it should not be framed as methodologically novel on the basis of this modeling choice alone.

    Ans: Thank you for this constructive comment. We agree that the original wording overstated the methodological novelty of the study. In the revised Discussion, we have removed the claim that the multilevel modeling approach is a unique contribution.
    Instead, we now emphasize that the principal contribution of the study lies in providing nationally representative evidence on the associations between physical bullying, verbal bullying, and cyberbullying and depressive symptoms among Bangladeshi adolescents. We also clarify that the multilevel modeling framework was adopted because it appropriately accounted for the hierarchical structure of the BAHWS data and contextual variation across clusters and districts. Consistent with the model comparison results, we acknowledge that the improvement in model fit over the two-level model was modest, while noting that the three-level model provided a statistically better fit and more appropriately reflected the survey design.
    These revisions provide a more balanced interpretation of the study's contribution and avoid overstating the methodological novelty of the multilevel modeling approach. The relevant changes have been incorporated into the Discussion (Page 20, Lines 502–13).

18. The limitations section is incomplete. In addition to the cross-sectional nature of the data and self-report bias, the authors should explicitly discuss the large reduction from 18,249 to 9,116 participants, the mismatch between bullying and depression time windows, and the lack of explicit suicide-specific measurement despite occasional discussion language that drifts in that direction.

    Ans: Thank you for this valuable suggestion. We agree that the limitations section should more comprehensively acknowledge the methodological constraints of the study. Accordingly, we have substantially expanded the Limitations section in the revised manuscript.
    Specifically, we now explicitly discuss: (i) the cross-sectional design and its inability to establish causal relationships; (ii) the use of self-reported measures and the potential for recall and reporting bias; (iii) the reduction in the analytical sample from 18,249 interviewed adolescents to 9,116 participants due to incomplete PHQ-9 outcome data, together with the po-

tential for selection bias and the inclusion of a participant flow diagram to clarify the sample selection process; (iv) the mismatch between the assessment periods of bullying victimization (previous 12 months) and depressive symptoms (previous two weeks), which limits temporal interpretation; and (v) the fact that the BAHWS did not collect information on suicide-specific outcomes, such as suicidal ideation or suicidal behaviors. Accordingly, we have revised the Discussion to ensure that the interpretation remains focused on depressive symptoms and does not extend to outcomes that were not measured.
These revisions provide a more complete and transparent discussion of the study limitations and their implications for interpreting the findings (Page 21, Lines 514–35).

Reviewer 2

1. In the Introduction, the term "bullying" and "harassment" are used interchangeably. The authors need to define each behavior.

   Ans: Thank you for this helpful comment. We agree that using the terms "bullying" and "harassment" interchangeably could lead to ambiguity. In response, we have carefully reviewed the manuscript and standardized the terminology throughout. Specifically, we now consistently use the term "bullying" to describe the exposure of interest, as this corresponds to the definitions and measures used in the Bangladesh Adolescent Health and Wellbeing Survey (BAHWS). References to "harassment" have been removed or replaced with "bullying," where appropriate, to ensure conceptual consistency and avoid confusion. These revisions have been incorporated throughout the Introduction and the remainder of the manuscript

2. More information regarding with marital status and education in Bangladesh is needed such as average age of marriage, the rate of the enrollment for higher education such as college or university. Cultural background affects the bullying behavior and depression.

   Ans: Thank you for this valuable suggestion. We agree that additional contextual information would help readers better understand the sociocultural setting of the study. Accordingly, we have expanded the Introduction to provide a brief overview of marital status, educational participation, and relevant cultural factors among Bangladeshi adolescents.
   Specifically, we now describe the legal minimum age of marriage in Bangladesh and note that early marriage among adolescent girls remains an important social issue in some settings. We also provide contextual information on educational participation during late adolescence, highlighting that many

adolescents either transition to higher education or discontinue their education because of financial constraints, early marriage, or employment. In addition, we discuss how cultural norms related to gender roles, family expectations, and social relationships may influence both bullying victimization and depressive symptoms. These additions provide a stronger rationale for including marital status and school/college attendance as explanatory variables in our analyses and better contextualize the study within the Bangladeshi setting. The relevant revisions have been incorporated into the Introduction (Page 04, Lines 81–93).

3. What percentage of adolescents who own internet devices? Information regarding with smartphones, PCs and other devices should be mentioned.

   Ans: Thank you for this valuable suggestion. We agree that providing additional context on adolescents' access to internet-enabled devices strengthens the background and interpretation of cyberbullying in the Bangladeshi context.
   In response, we have revised the introduction to include information on the increasing use of internet-enabled devices among Bangladeshi adolescents and its relevance to cyberbullying. We have also clarified in the Methods and Results that internet device use was included as an explanatory variable in the multilevel analyses to account for adolescents' access to digital technologies. Furthermore, the distribution of internet device use among the study participants is now presented in Table 1, allowing readers to understand the proportion of adolescents with access to internet-enabled devices in the analytical sample.

4. On line 57 to 78 should be more organized. In line 57 to 67, many countries and many regions are written. Need to be written separately and need to be structured.

   Ans: Thank you for this helpful suggestion. We agree that the original Introduction would benefit from a clearer and more logical organization. Accordingly, we have substantially revised and restructured the Introduction to improve its coherence and readability.
   Specifically, the section describing the global epidemiology of adolescent depression has been reorganized into a more logical sequence. We first present the global burden of adolescent depression, followed by evidence from high-income countries and then low- and middle-income countries. We also condensed repetitive descriptions, improved the transitions between paragraphs, and reorganized the subsequent sections to provide a clearer progression from the global context to the South Asian and Bangladeshi context, culminating in the research gap and study objective. These revi-

sions improve the overall flow of the Introduction and provide a stronger rationale for the present study.
The revised text has been incorporated on Page 3, Lines 38–49.

5. On 146 to 157, the description needs to be divided in paragraphs.

   Ans: Thank you for this helpful suggestion. We agree that the original presentation of this section was too dense and could be improved for readability. Accordingly, we have reorganized the text by dividing the content into separate paragraphs according to the different methodological components. This revision improves the logical flow, readability, and clarity of the section without altering its scientific content.

6. In the survey, how the victimizations of bullying were assessed? Since the percentage of the bullying depend much on the expression of the question, the authors need to clarify the questions. Is the frequency assessed? Intensity might affect the mental health. More precise information is needed.

   Ans: Thank you for this important comment. We agree that a clearer description of the bullying measures improves the transparency and reproducibility of the study. Accordingly, we have added a new subsection entitled "Bullying experiences" in the Methods.
   In the revised manuscript, we now describe how bullying victimization was assessed in the BAHWS, including the three bullying domains (physical bullying, verbal bullying, and cyberbullying), the recall period, and the coding of each bullying variable used in the analyses. We also clarify that the survey recorded whether adolescents experienced each type of bullying but did not collect detailed information on the frequency, duration, or severity (intensity) of bullying victimization. Consequently, our analyses were based on the occurrence of each bullying type rather than its frequency or intensity. To complement these analyses, we additionally created a composite bullying variable representing the number of bullying types experienced (none, one, two, or three) and examined its association with depressive symptoms in an additional multilevel analysis.
   These revisions have been incorporated into the Methods section (Page 7-8, Lines 191–213)

7. Some of the adolescents experience multiple types of bullying. The authors need to conduct further analysis and clarify the following results; percentage of the adolescents who have been victimized of multiple types of bullying, how the victimization of multiple types of victim associate with the degree of depression.

   Ans: Thank you for this valuable suggestion. We agree that examining the cumulative experience of bullying provides additional insight into its

association with depressive symptoms.
In response, we created a composite bullying exposure variable based on the number of bullying types experienced by each adolescent. Participants were classified into four mutually exclusive categories: no bullying, one type of bullying, two types of bullying, and three types of bullying. We first summarized the weighted prevalence of each category to describe the distribution of multiple bullying victimization in the study population.
We then conducted an additional three-level linear mixed-effects analysis using this composite bullying variable as the primary exposure while adjusting for the same covariates included in the main analyses. The results are presented in the newly added Table 5, which demonstrates the association between cumulative bullying victimization and PHQ-9 depression scores. This additional analysis provides a more comprehensive assessment of the relationship between exposure to multiple forms of bullying and depressive symptoms and directly addresses the reviewer's concern.
The Methods, Results, Discussion, and the new Table 5 have been added to the revised manuscript (Page 17, Lines 389–96).

8. Some of the descriptions are arbitrary. For example, on line 342 the authors state as follows; "Our findings confirmed that bullying victimization is strongly associated with poor adolescent mental health outcomes, particularly depression." How did the authors conclude that the association is "strong"? Some of the CIs are not as high as could be judged as "strong", On line 354, the authors state that "Adolescents exposed to bullying consistently reported higher depression levels, a result in line with international evidence that highlights bullying as a major risk factor for adolescent mental health problems." However, whether the adolescents exposed to bullying "consistently" is not assessed in the questionnaire. At least, it is not written in the method.

   Ans: Thank you for this thoughtful comment. We agree that some of the wording in the Discussion overstated the interpretation of our findings. Accordingly, we have carefully revised the Discussion to use more precise language that is consistent with the study design and the available data.
   Specifically, we replaced terms such as "confirmed" and "strongly associated" with more appropriate expressions such as "showed significant associations" or "were associated." We also removed the term "consistently reported," as the BAHWS assessed whether adolescents experienced each form of bullying during the specified recall period but did not measure the frequency or consistency of bullying victimization. In addition, we revised several statements to avoid causal language and ensured that references to depression and bullying accurately reflect the observed associations rather than implying causal effects or unsupported effect magnitudes.
   These revisions have been incorporated throughout the Discussion (Page

18, Lines 416–439).

9. The paragraph of the Discussion part is too long. The discussion of the two hypotheses is included in one paragraph2. It should be written separately.

   Ans: Thank you for this helpful suggestion. We agree that the original Discussion contained several lengthy paragraphs that reduced readability and combined the interpretation of multiple findings. In response, we have comprehensively reorganized the Discussion section by dividing long paragraphs into shorter, more focused sections.
   Specifically, the Discussion has been restructured so that the findings related to each research hypothesis are interpreted separately. In addition, we have reorganized the remaining sections to discuss the influence of sociodemographic characteristics, the implications of the findings, the strengths and limitations of the study, and the conclusion in separate paragraphs. These revisions improve the logical flow, readability, and clarity of the Discussion while providing a more focused interpretation of the study findings.

10. On Line 343-344, the authors state ". Our findings confirmed that bullying victimization is strongly associated with poor adolescent mental health outcomes, particularly depression." However, the authors did not examine mental health outcomes other than depression. More precise description is needed.

    Ans: Thank you for this important observation. We agree that the original wording was imprecise because the present study specifically examined depressive symptoms, rather than broader mental health outcomes. Accordingly, we have revised the text throughout the manuscript to ensure that the interpretation accurately reflects the measured outcome. Specifically, the statement has been revised to refer to the association between bullying victimization and depressive symptoms (PHQ-9 depression score) rather than general mental health outcomes. We also carefully reviewed the Introduction, Results, Discussion, and Conclusion to replace broad references to "mental health" or "mental well-being" with more precise terminology where appropriate, ensuring consistency with the study objective, outcome measure, and statistical analyses. The revised text has been incorporated into the Discussion.

11. Line 393 to 410 is the repletion of the Results. The insights from the results should be described in the Discussion.

    Ans: Thank you for this constructive comment. We agree that parts of the original Discussion overly repeated the statistical results rather than

providing interpretation. Accordingly, we have substantially revised this section to focus on the implications of the findings instead of restating the numerical results.
Specifically, we have reorganized the paragraph to emphasize the potential mechanisms underlying the observed associations between sociodemographic characteristics and depressive symptoms and to interpret these findings in the context of previous literature. We now discuss how socioeconomic disadvantage, age, school attendance, gender inequality, and early marriage may contribute to adolescents' vulnerability to depressive symptoms, rather than reiterating the regression results. We also removed repetitive descriptions, eliminated the statement regarding hypothesis acceptance, and ensured that the discussion remains focused on interpreting the findings rather than repeating the Results section.
These revisions improve the scientific interpretation, readability, and overall flow of the Discussion and have been incorporated into the revised manuscript (Page 19, Lines 448-78).

General: We thank all reviewers for their constructive suggestions and comments to improve the quality of the paper.

Yours sincerely,

Ruhul Amin

Academic Editor

Plos Mental Health
