# Supplementary material for: Exploring the association between bullying and depression symptoms among adolescents in Bangladesh: Findings from nationally representative survey
Source: PLOS Ment Health. 2026 Oct 28;3(10):e0000722. doi: 10.1371/journal.pmen.0000722 (PMC13716191; doi:10.1371/journal.pmen.0000722)
Supplement: Supplementary file 3 [file pmen.0000722.s009.pdf]

Ruhul Amin
Research Associate
Bangladesh Institute of Governance and Management
Dhaka-1207, Bangladesh
E-mail: ruhul.aminbigm.edu.bd

August 24, 2026

Academic Editor
Plos Mental Health

Dear Dr. Helena R. Slobodskaya,

Please find the revised version of the manuscript (Submission ID: PMEN-D-26-00157R1 entitled:**"Exploring the Association Between Bullying and Depression Symptoms Among Adolescents in Bangladesh: Findings from Nationally Representative Survey"**, and consider it for publication in the journal **Plos Mental Health**.

We have revised the manuscript according to the reviewer's comments and suggestions.

Authors' Note: The major corrections and new addendum are highlighted in magenta color texts for Editorial Board Member(EBM), red color texts for Reviewer 1 and teal color texts for Reviewer 2 in the revised manuscript. Additional corrections are highlighted in **a different color** following the journal template.

Legend (text colors for revision and corrections)

- EBM
- Reviewer 1
- Reviewer 2

EBM

1. Linear mixed models: Please, indicate in Statistical Analysis if you tested two models in the total sample: 1) with the three bullying variables and 2) with the composite bullying. It is not clear whether the same two models (without gender and marital status) were also tested in the three subgroups and if not, why.

   Ans: Thank you for highlighting this lack of clarity. We have revised the

Statistical Analysis subsection to explicitly describe the sequence and specification of the linear mixed-effects models.

Following selection of the appropriate multilevel structure, two models were fitted in the total sample. The first model simultaneously included the three specific bullying variables (physical, verbal, and cyberbullying), together with the prespecified sociodemographic covariates and bullying × gender and bullying × marital status interaction terms. The second model replaced the three specific bullying variables with the composite bullying measure (none, one, two, or three types) to examine the cumulative association between multiple bullying exposures and depressive symptoms.

In addition, separate adjusted models containing the three specific bullying variables simultaneously were fitted for ever-married females, unmarried females, and unmarried males. Gender and marital status were not included as covariates in these subgroup-specific models because they define the respective subgroups and therefore provide no meaningful within-group variation. These analyses are now explicitly described as exploratory subgroup analyses, while between-group heterogeneity was formally evaluated through bullying × gender and bullying × marital status interaction terms in the pooled model.

The composite bullying model was not repeated within each subgroup, because its purpose was to evaluate the overall cumulative association between exposure to multiple bullying types and depressive symptoms in the total sample. The subgroup analyses instead focused on the associations of the three specific forms of bullying within each demographic group. This analytical strategy and its rationale have now been clarified in the revised Statistical Analysis subsection (Page 09-10, Lines 248–251).

2. Results, 1st para and Table 1: Please check the figures for gender-by-marital status groups, they do not sum up correctly: if there were 2470 (27%) married females and no married males, why total number of married participants was 6646 (73%)? Reviewer 1 noted other substantive discrepancies that need to be addressed.

   Ans:Thank you for identifying this inconsistency. We carefully rechecked the marital-status and gender–marital status variables against the analytic dataset and confirmed that the marital-status categories in Table 1 had been inadvertently reversed during table preparation. Because the BAHWS analytic sample includes ever-married females but no ever-married males, the proportion of ever-married participants should correspond to the ever-married female subgroup. Accordingly, 27.25% of the analytic sample

were ever-married and 72.75% were unmarried, consistent with the gender–marital status distribution.

We have corrected the marital-status frequencies, percentages, and corresponding narrative in Table 1 and the first paragraph of the Results (Page 12, Lines 323-335). The revised gender–marital status distribution is also reported consistently as 27.25% ever-married females, 42.25% unmarried females, and 30.51% unmarried males.

In response to this comment and the related concerns raised by Reviewer 1, we also conducted a complete audit of the descriptive statistics and multivariable results, including frequencies, weighted percentages, regression coefficients, interaction terms, and their interpretations across the Abstract, Results, Tables, Discussion, and Conclusion. All identified discrepancies have been reconciled with the final analytic output.

3. Regarding comparisons between 2- and 3-level mixed models, I was not able to find the Preliminary analysis subsection in the manuscript.

   Ans: Thank you for pointing this out. We apologize for the lack of clarity in the previous revision. Although the comparison between the two-level and three-level linear mixed-effects models was included in the manuscript, it was not presented under a clearly identifiable "Preliminary analysis" subsection.

   In the revised manuscript, we have now created a separate subsection entitled "Preliminary analysis and selection of the multilevel model" in the Results section. This subsection presents the comparison of the two- and three-level models using the variance components, AIC, AICc, BIC, ICC, −2 log-likelihood, and likelihood-ratio test. Based on these model-fit criteria, the three-level model was retained for the subsequent multivariable analyses. This reorganization clearly separates the model-selection procedure from the primary findings concerning bullying and depressive symptoms (Page 15-16, Lines 377–390; Table 3).

Reviewer 1

1. The operationalization of "bullying" is a central concern: The measures appear to capture any physical violence, verbal/social mistreatment, or online harassment by "anyone," and a single occurrence appears sufficient for classification. This does not necessarily meet the usual definition of bullying involving repetition and a power imbalance. The authors should either justify these measures as bullying or, more appropriately, relabel the exposures as violence/harassment throughout and reconsider the framing of the paper.

Ans: We sincerely appreciate the reviewer's thoughtful concern regarding the operationalization of bullying. We respectfully maintain that our use of the term "bullying" is appropriate and aligned with the measurement framework of the Bangladesh Adolescent Health and Wellbeing Survey (BAHWS) 2019–20. The BAHWS is a standardized national survey that specifically assessed adolescents' experiences of physical, verbal/social, and cyberbullying as distinct forms of bullying. In the present study, we did not develop or impose an independent definition of bullying; rather, we followed the survey's established operationalization and used the bullying-related variables as they were defined and collected in the original questionnaire.

2. The large reduction in the analytic sample remains insufficiently resolved: Only 9,116 of 19,888 identified adolescents contributed complete PHQ-9 data. Given that different questionnaire modules were administered across cluster types, the authors should clarify whether this reflects true missingness or the survey design itself. This distinction is critical for selection bias, weighting, and the claim that the analytic results are nationally representative.

   Ans: Thank you for this important comment. We agree that the reduction in the analytic sample required clearer explanation. The BAHWS 2019–20 identified 19,888 adolescents aged 10–19 years. However, the PHQ-9 depression module was administered only to adolescents in household survey clusters where the mental health module was included as part of the survey design. Therefore, the reduction to the eligible sample primarily reflects planned questionnaire administration, rather than conventional missing data due to participant nonresponse. After restricting the analysis to adolescents eligible for the PHQ-9 module and excluding the small number of incomplete responses within that eligible group, the final analytic sample comprised 9,116 adolescents.

3. There are several substantive inconsistencies across the reported results that require a complete audit: For example, Table 1 reports 72.75% as married, whereas the gender–marital categories indicate that 27.25% were married females; the abstract describes ever-married status as associated with higher depressive symptoms although marital status is non-significant in the adjusted model; and the abstract reports a significant verbal-bullying × gender interaction whereas Table 4 reports this interaction as non-significant. These discrepancies substantially undermine confidence in the current results and should be reconciled throughout.

   Ans:Thank you for identifying these important inconsistencies. We agree

that discrepancies across the Abstract, Results, tables, Discussion, and Conclusion could lead to confusion regarding the interpretation of the findings. In response, we conducted a complete audit of the reported descriptive statistics, regression estimates, interaction results, and their corresponding interpretations and reconciled these throughout the revised manuscript.

First, the marital-status categories in Table 1 were corrected. The revised table now consistently reports 27.25% of adolescents as ever-married and 72.75% as unmarried, in agreement with the gender–marital status categories. The corresponding descriptive text has also been corrected (Table 1; Results, Page 12, Lines 327).

Second, we revised the Abstract to distinguish the unadjusted descriptive findings from the adjusted multivariable results. Although ever-married adolescents had higher mean depressive symptom scores in the descriptive analysis, marital status was not independently associated with depressive symptoms in the fully adjusted model. The previous statement implying an independent adjusted association with marital status has therefore been removed (Abstract, Page 2, Lines 23–26).

Third, we corrected the reporting and interpretation of the interaction analyses throughout the manuscript. The final model showed significant effect modification by gender only for physical bullying, whereas the gender interactions for verbal and cyberbullying were not statistically significant. Furthermore, none of the bullying × marital status interactions were statistically significant. The Abstract, Results, Discussion, and Conclusion have been revised accordingly (Pages 17-18, Lines 406–408; Table 4).

Finally, the subgroup analyses of ever-married females, unmarried females, and unmarried males are now explicitly described as exploratory. Differences in the magnitude or statistical significance of subgroup-specific estimates are no longer interpreted as evidence of formal between-group differences. Instead, conclusions regarding heterogeneity are based on the formal bullying × gender and bullying × marital status interaction tests. Accordingly, Hypothesis 2 is now interpreted as partially supported, with evidence of gender-related effect modification for physical bullying only and no evidence of effect modification by marital status for any bullying type (Discussion, Page 20-21, Lines 485–512).

Following this audit, all relevant values and interpretations in the Abstract, Table 1, Table 4, Results, Discussion, and Conclusion have been revised to ensure consistency with the final statistical analyses.

4. The claims regarding differences by sex and marital-status group exceed the formal analyses presented: Separate significant associations within ever-married females, unmarried females, and unmarried males do not demonstrate that associations differ between these groups. If variation by marital status is a central hypothesis, this should be tested formally through appropriate interaction terms; otherwise, the subgroup findings should remain descriptive/exploratory and conclusions regarding between-group differences should be substantially tempered.

   Ans: Thank you for this important methodological comment. We agree that statistically significant associations observed separately within adolescent subgroups do not, by themselves, demonstrate that the associations between bullying and depressive symptoms differ significantly across groups. Because both gender and marital status are central to our subgroup analyses, we agree that potential effect modification should be evaluated through formal interaction tests.

   Accordingly, in the revised primary multivariable three-level linear mixed-effects model, we retained gender and marital status as separate explanatory variables, consistent with the recommendation from the first round of review. To formally assess effect modification, we examined interactions between each bullying type and gender and between each bullying type and marital status. The interaction analyses identified significant effect modification by gender only for physical bullying , whereas the gender interactions for verbal and cyberbullying were not statistically significant. No significant effect modification by marital status was observed for any bullying type .

   We did not additionally include the combined gender–marital status subgroup variable in the primary model because it is derived directly from gender and marital status, which were already included as explanatory variables. Simultaneously including the separate variables and their combined grouping would introduce redundant parameterization and complicate model estimation and interpretation. Retaining gender and marital status separately also allowed their main effects and their respective interactions with each bullying type to be evaluated directly.

   Accordingly, the analyses of ever-married females, unmarried females, and unmarried males are now explicitly presented as complementary exploratory subgroup analyses rather than formal evidence of between-group differences. The revised manuscript no longer interprets differences in subgroup-specific statistical significance as evidence that the associations differ significantly between groups. Instead, conclusions regarding heterogeneity are based on the formal bullying × gender and bullying × marital status interaction tests. Consequently, Hypothesis 2 is interpreted as partially supported,

as the association between physical bullying and depressive symptoms varied significantly by gender, whereas no significant gender-specific variation was observed for verbal or cyberbullying, and marital status did not significantly modify the association for any bullying type. The Methods, Results, Discussion, and Conclusion have been revised accordingly.

Reviewer 2

1. The authors stated as follows: "Although the legal minimum age of marriage is 18 years for females and 21 years for males, the median age at first marriage among women remains 16.9 years, and approximately 51.4% of women aged 20–24 years were 84 married before the age of 18, indicating that child marriage remains common despite legal 85 restrictions." It is restricted by the law, how the adolescents get married? What is the definition of marriage in this paper? Explanations cause confusion, thus the authors need to add more explanation on the cultural and societal background. Moreover, the authors need to elucidate socio-economical characteristics that early-married adolescents have. These background associate with the victimization.

   Ans: Thank you for this important comment. We agree that our previous description did not sufficiently explain the apparent discrepancy between the statutory minimum age of marriage and the occurrence of marriage during adolescence in Bangladesh, nor did it adequately define the marital-status classification used in this study. We have therefore expanded the sociocultural and socioeconomic context of adolescent marriage in the introduction.

   Specifically, we now clarify that, although the statutory minimum age of marriage is 18 years for females and 21 years for males, marriage before age 18 continues to occur in Bangladesh. We further describe adolescent marriage within the broader context of educational discontinuation, socioeconomic circumstances, family expectations, and gender-related social norms. Consistent with the BAHWS 2019–20, more than three-fourths of ever-married female adolescents lived in rural areas, only 25% had completed secondary education or higher, and 83% had dropped out of school. These characteristics provide important social context because they may also be related to adolescents' exposure and vulnerability to interpersonal victimization and depressive symptoms.

   We have also clarified the operational definition of marital status in the Methods section . Following the BAHWS 2019–20 classification, female adolescents aged 15–19 were categorized as ever-married or unmarried, whereas the male adolescent sample included unmarried males only. Among the ever-married female adolescents surveyed, 97% were currently married.

Accordingly, the term "ever-married" in our manuscript reflects the survey-defined demographic classification and should not be interpreted as an assessment of the legal validity of individual marriages.

Finally, we have revised the relevant interpretation to avoid suggesting that marital status itself causes bullying victimization. Instead, marital status is considered within the broader socioeconomic and sociocultural circumstances that may be associated with both victimization and depressive symptoms. These revisions have been incorporated in the Introduction (Page 4, Lines 84–101) and Methods section (Page 08, Lines 206–212).

2. The authors added analysis shown in Table5 based on the reviewer's comment. The authors should include the hypothesis regarding with this analysis in the Introduction part. Moreover, the authors need to describe the effect of victimization of multiple types of bullying in Discussion part.

   Ans: Thank you for this valuable comment. We agree that the analysis of cumulative exposure to multiple forms of bullying presented in Table 5 should be supported by an explicit hypothesis in the Introduction and further interpreted in the Discussion. Accordingly, we have added a new hypothesis proposing that exposure to a greater number of bullying types is associated with progressively higher depressive symptom scores.

   We have also expanded the Discussion to interpret the composite bullying findings. In the adjusted model, adolescents exposed to one, two, and three types of bullying had progressively higher depressive symptom scores compared with those reporting no bullying (β = 1.25, 2.61, and 4.99, respectively; all $p < 0.001$). We now discuss this graded pattern as evidence of a cumulative or dose–response association, suggesting that exposure to multiple forms of victimization may compound psychological distress rather than each bullying experience operating in isolation.
   These revisions have been incorporated into the Introduction (Page 06, Lines 152–153) and Discussion (Page 21, Lines 513-522).

Figure Resubmissions

1. While revising your submission, we strongly recommend that you use PLOS's NAAS tool (https://ngplosjournals.pagemajik.ai/artanalysis) to test your figure files. NAAS can convert your figure files to the TIFF file type and meet basic requirements (such as print size, resolution), or provide you with a report on issues that do not meet our requirements and that NAAS cannot fix.

After uploading your figures to PLOS's NAAS tool - https://ngplosjournals.pagemajik.ai/artanalysis, NAAS will process the files provided and display the results in the "Uploaded Files" section of the page as the processing is complete. If the uploaded figures meet our requirements (or NAAS is able to fix the files to meet our requirements), the figure will be marked as "fixed" above. If NAAS is unable to fix the files, a red "failed" label will appear above. When NAAS has confirmed that the figure files meet our requirements, please download the file via the download option, and include these NAAS processed figure files when submitting your revised manuscript.

Ans: Thank you for this helpful guidance. We have carefully followed the PLOS figure submission requirements and used the PLOS NAAS tool to assess and process all figure files. The figures were checked for the required technical specifications, including file format, resolution, and print dimensions. Where necessary, the figure files were processed through NAAS to ensure compliance with the journal's requirements. The NAAS-processed figure files have been downloaded and incorporated into the revised submission. We have therefore replaced the original figure files with the validated, NAAS-processed versions in accordance with the journal's instructions.

General: We thank all reviewers for their constructive suggestions and comments to improve the quality of the paper.

Yours sincerely,

Ruhul Amin

Academic Editor

Plos Mental Health
