# Supplementary material for: Exploring the association between bullying and depression symptoms among adolescents in Bangladesh: Findings from nationally representative survey
Source: PLOS Ment Health. 2026 Oct 28;3(10):e0000722. doi: 10.1371/journal.pmen.0000722 (PMC13716191; doi:10.1371/journal.pmen.0000722)
Supplement: Supplementary file 4 [file pmen.0000722.s010.pdf]

Ruhul Amin
Research Associate
Bangladesh Institute of Governance and Management
Dhaka-1207, Bangladesh
E-mail: ruhul.aminbigm.edu.bd

September 26, 2026

Academic Editor
Plos Mental Health

Dear Dr. Helena R. Slobodskaya,

Please find the revised version of the manuscript (Submission ID: PMEN-D-26-00157R1 entitled:**"Exploring the Association Between Bullying and Depression Symptoms Among Adolescents in Bangladesh: Findings from Nationally Representative Survey"**, and consider it for publication in the journal **Plos Mental Health**.

We have revised the manuscript according to the reviewer's comments and suggestions.

Authors' Note: The major corrections and new addendum are highlighted in magenta color texts for Editorial Board Member(EBM), red color texts for Reviewer 1 and teal color texts for Reviewer 2 in the revised manuscript. Additional corrections are highlighted in **a different color** following the journal template.

Legend (text colors for revision and corrections)

- EBM
- Reviewer 1
- Reviewer 2

EBM

1. Linear mixed models. I was not able to find the description of your analytical strategy and its rationale on Pages 09-10, Lines 248–251. It is still unclear why the model with the composite bullying was not tested in the three subgroups. Please explain this in Statistical Analysis subsection.

   Ans: Thank you for highlighting this issue. We apologize for the incor-

rect page and line numbers provided in our previous response. We have revised the Statistical Analysis subsection to clarify the analytical strategy and rationale for not repeating the composite bullying model within the three subgroups. Specifically, the composite bullying model was fitted to the total sample to assess the overall cumulative association between multiple forms of bullying victimization and depressive symptoms, whereas subgroup analyses focused on the associations of the three specific bullying forms across demographic groups. This clarification has been added to the revised Statistical Analysis subsection (Page 10, Lines 278–89).

2. Please address Reviewer 2 concern about the reliability of the data on married adolescents. It would be helpful to describe how marriage procedures for those under the statutory minimum age differ from the standard marriage procedures. The possibility of mixing domestic violence with bullying should also be addressed and clearly acknowledged as a limitation.

   Ans:Thank you for this important comment. We have revised the manuscript to address both concerns. First, in the Data Source and Study Population section, we have clarified that the statutory minimum age for marriage in Bangladesh is 18 years for females and 21 years for males. We further explain that the Child Marriage Restraint Act, 2017 includes a special provision for marriage below the statutory age under specified circumstances, including consideration of the minor's best interests, court direction, and parental or guardian consent. The BAHWS, however, did not collect information on the legal registration or procedures through which individual adolescent marriages occurred, including whether marriages below the statutory age followed this provision. Therefore, "ever-married" is interpreted as the survey-reported marital-status classification rather than verification of the legal procedure or statutory compliance of individual marriages (Page 06-07, Lines 158–70). Second, we have clarified that the BAHWS bullying/violence measures were not restricted to peer perpetrators. Among ever-married females, reported perpetrators included husbands, in-laws, and other family members. Consequently, some experiences classified as physical violence or verbal/social bullying may overlap with intimate partner or domestic violence. Because these experiences could not be disentangled in this secondary analysis, we now explicitly acknowledge this potential overlap as a limitation and caution that the findings among ever-married females should not be interpreted as reflecting peer bullying exclusively (Page 22-23, Lines 574–82).

3. In response to Reviewer 2 comment # 1 about marriage, you added a new paragraph to the Introduction (Page 4-5, lines 84-101). However, this text largely repeats the next paragraph on Page 5, lines 102-116. Please, edit the text carefully to avoid repetitions

and to improve the precision and coherence of the manuscript.

Ans: Thank you for highlighting this issue. We apologize for the repetition between the two paragraphs. To improve the precision and coherence of the Introduction, we have deleted the subsequent paragraph (Page 5, Lines 102–116) and retained the newly added paragraph (Page 4–5, Lines 84–101). This revision eliminates the overlapping content while preserving the relevant contextual discussion.

Reviewer 1

1. No further comments

   Ans: Thank you for reviewing our revised manuscript and for your valuable comments throughout the review process. We appreciate your time and consideration.

Reviewer 2

1. Marriage is governed by law. Although the law prohibits women under the age of 18 from marrying, it is not clearly specified how marriage procedures are carried out in such cases.This is an important point that affects the reliability of the data, but the authors do not explain it. Moreover, in the survey, by whom the respondents are bullied. If any of the respondents are married, domestic violence may be included in the "bullied"participants. For this reason, the data can be considered unreliable.

   Ans: Thank you for raising these important concerns. We have substantially clarified both issues in the revised manuscript. First, the BAHWS classified adolescents according to their reported marital status but did not collect information on the legal registration or circumstances through which individual adolescent marriages occurred. Therefore, as a secondary analysis, our study cannot determine how marriages involving respondents below the statutory minimum age were conducted. We have now explicitly acknowledged this issue in the Limitations section (Page 22-23, Lines 569–82). Second, we agree that the BAHWS-defined bullying/violence measures were not restricted to peer-perpetrated experiences. The BAHWS collected information on perpetrators and reported that, among ever-married females, husbands, in-laws, and other family members were among the perpetrators of physical violence and verbal/social bullying. For example, among ever-married females reporting verbal/social bullying, 47.0% identified husbands and 37.3% identified in-laws as perpetrators (multiple responses were possible). Therefore, some experiences categorized as physical violence or verbal/social bullying may overlap conceptually with

intimate partner or domestic violence and should not be interpreted exclusively as peer bullying. We have consequently clarified the definition and interpretation of these exposures in the Methods section (Page 06-07, Lines 158–70; page: 9, lines: 232-41) and explicitly acknowledged this potential overlap in the Limitations section. Because the present study is a secondary analysis, we retained the original BAHWS operational definitions rather than retrospectively reclassifying individual experiences according to perpetrator. The revised manuscript therefore interprets these measures as BAHWS-defined bullying/violence experiences rather than exclusively peer-perpetrated bullying.

Yours sincerely,

Ruhul Amin

Academic Editor

Plos Mental Health
