# Supplementary material for: Exploring the association between bullying and depression symptoms among adolescents in Bangladesh: Findings from nationally representative survey
Source: PLOS Ment Health. 2026 Oct 28;3(10):e0000722. doi: 10.1371/journal.pmen.0000722 (PMC13716191; doi:10.1371/journal.pmen.0000722)
Supplement: Supplementary file 5 [file pmen.0000722.s011.pdf]

Ruhul Amin
Research Associate
Bangladesh Institute of Governance
and Management
Dhaka-1207, Bangladesh
E-mail: ruhul.aminbigm.edu.bd

October 5, 2026

Academic Editor
Plos Mental Health

Dear Dr. Helena R. Slobodskaya,

Please find the revised version of the manuscript (Submission ID: PMEN-D-26-00157R1 entitled:**"Exploring the Association Between Bullying and Depression Symptoms Among Adolescents in Bangladesh: Findings from Nationally Representative Survey"**, and consider it for publication in the journal **Plos Mental Health**.

We have revised the manuscript according to the editor's comments and suggestions.

Authors' Note: The major corrections and new addendum are highlighted in magenta color texts for Editorial Board Member(EBM) in the revised manuscript.

Legend (text colors for revision and corrections)

- EBM

EBM

1. Please define the abbreviation BAHWS when first introduced on p. 4, line 90 (Adolescent Mental `Wellbeing_Marked_RA_V4_with` track changes).

   Ans: Thank you for pointing this out. We have now defined BAHWS at its first occurrence in the Introduction as the "Bangladesh Adolescent Health and Wellbeing Survey (BAHWS) 2019–20." The abbreviation BAHWS is used consistently thereafter (Page 4, Line 90-91).

2. The statutory minimum age for marriage is presented twice: in the Introduction on p. 4, line 86, and in the Data Sources subsection on p. 7, line 179. Please remove the repetition.

Ans:Thank you for identifying this repetition. We have removed the statement regarding the statutory minimum age for marriage from the Introduction and retained it in the Data Sources subsection, where it is directly relevant to the definition and interpretation of the "ever-married" category and is accompanied by the appropriate legal context. The Introduction has been revised accordingly to focus on the broader social context of adolescent marriage in Bangladesh (Page 4, Lines 85–88).

3. The BAHWS is also described twice: in the Introduction on p. 5, lines 130-133, and in the Data Sources subsection on p. 7, lines 164-170. I would suggest to present all details of the study design in the Data Sources subsection. In the Introduction the Authors could briefly indicate the hierarchical nature of the survey data.

   Ans: Thank you for this helpful suggestion. We agree and have revised the manuscript to avoid repetition. Specifically, we have removed the detailed description of the BAHWS sampling design from the Introduction and retained the complete description of the survey design and sampling procedures in the Data Sources subsection. In the Introduction, we now briefly indicate only the hierarchical nature of the BAHWS data, with adolescents clustered within PSUs and districts, and explain its relevance to the use of multilevel modelling. This revision improves the organization of the manuscript while retaining the methodological rationale for the three-level analytical approach (Page 5, Lines 116–22).

4. A linear mixed model is described in the first paragraph of Statistical Analysis on p.10, and then in a separate subsection on pp. 12-13. I would suggest to place the Linear Mixed Model subsection after the sentence "Since the study employed a multilevel data structure, we utilized a linear mixed model to identify the significant determinants that addressed the within-group correlation [31,32]". It would be helpful to place the text about criteria used for model selection (on p. 10, lines 285-288) at the end of the Linear Mixed Model subsection.

   Ans: Thank you for this helpful suggestion. We have reorganized the Statistical Analysis section accordingly. The Linear Mixed Model subsection has now been moved immediately after the sentence introducing the use of a linear mixed-effects model for the hierarchical survey data. This allows the three-level model structure, estimation procedure, incorporation of sampling weights, and model assumptions to be described directly after the analytical approach is introduced. We have also moved the description of the criteria used to compare the two-level and three-level models to the end of the Linear Mixed Model subsection. This revised organization provides a more coherent progression from the rationale for multilevel

modelling, through model specification and estimation, to model selection and subsequent adjusted analyses. No analytical procedures or results were changed; this revision concerns the organization and clarity of the Methods section.

5. In Statistical Analysis, subgroup analyses is presented four times: at the end of the first paragraph on p. 10, lines 83-84; in the second paragraph on p. 11, lines 294-300 and 305-307, and in the third paragraph on pp. 11-12, lines 318-326. I would suggest to describe each of the the three models tested after the subsection on the Linear Mixed Model in the same order as they are presented in the Results: (1) the main model with three bullying variables in the total sample, (2) the main model with three bullying variables in the three subgroups and (3) the additional model with composite bullying in the total sample.

   Ans: Thank you for this helpful suggestion. We have reorganized the Statistical Analysis section to eliminate the repeated descriptions of the subgroup analyses and improve the logical flow of the analytical methods. The description of the linear mixed-effects model has now been integrated with the preceding methodological text rather than presented as a separate subsection. The model-selection criteria are presented immediately after the description of the multilevel model. Subsequently, the three adjusted analyses are described once and in the same order as presented in the Results: (1) the primary model in the total sample, including physical, verbal, and cyberbullying and their interactions with gender and marital status; (2) separate exploratory models for ever-married females, unmarried females, and unmarried males, including the three bullying variables; and (3) an additional model in the total sample using the composite bullying measure to assess the cumulative association of exposure to multiple forms of bullying with depressive symptoms. The previously repeated descriptions of the subgroup analyses have been removed. These revisions improve the clarity and consistency of the Statistical Analysis section without changing the analyses or results(page: 9-11, lines:254-313).

6. Please explain how the coefficients for each value of the composite bullying measure (Table 5) were calculated.

   Ans: Thank you for this comment. We have clarified the estimation and interpretation of the coefficients for the composite bullying measure in the Statistical Analysis section. Specifically, the composite measure was entered into the total-sample linear mixed-effects model as a categorical variable with four levels (none, one, two, or three bullying types), with no bullying exposure as the reference category. Accordingly, the coefficients reported in Table 5 for one, two, and three bullying types represent adjusted mean

differences in depressive symptom scores relative to the no-bullying group. We have also clarified the reference category in Table 5 footnote(page:11, lines: 306-313).

7. In the Results, the selection of the multilevel model subsection is presented after the subsection "Descriptive characteristics and distribution of depressive symptoms". Therefore, it would be helpful to remove "Preliminary analysis" from the subtitle.

   Ans: Thank you for this helpful suggestion. We agree that the term "Preliminary analysis" is unnecessary given the placement of this subsection after the descriptive results. Accordingly, we have revised the subsection title from "Preliminary analysis and selection of the multilevel model" to "Selection of the best-fitting multilevel model." No changes were made to the analyses or results presented in this subsection.

8. I would suggest to describe strengths and limitations in a separate subsection after Discussion and before the Conclusions.

   Ans: Thank you for this helpful suggestion. We have reorganized the manuscript accordingly. The strengths and limitations, which were previously presented within the Discussion, have now been moved to a separate subsection entitled "Strengths and limitations," positioned after the Discussion and immediately before the Conclusions. This revision improves the organization and clarity of the manuscript without changing the substantive content.

Yours sincerely,

Ruhul Amin

Academic Editor

Plos Mental Health
