# Supplementary material for: Exploring the association between bullying and depression symptoms among adolescents in Bangladesh: Findings from nationally representative survey
Source: PLOS Ment Health. 2026 Oct 28;3(10):e0000722. doi: 10.1371/journal.pmen.0000722 (PMC13716191; doi:10.1371/journal.pmen.0000722)
Supplement: Supplementary file 6 [file pmen.0000722.s001.docx]

Exploring the Association Between Bullying and Depression Symptoms Among Adolescents in Bangladesh: Findings from Nationally Representative Survey

Table S1: Excluded adolescent sociodemographic information

| Variable Name | Attribute | Frequency (%) | Weighted Percentage |
| --- | --- | --- | --- |
| Age | 15 | 1815(19.87) | 19.83 |
|  | 16 | 2065(22.61) | 22.70 |
|  | 17 | 1933(21.17) | 21.30 |
|  | 18 | 1869(20.46) | 20.69 |
|  | 19 | 1451(15.89) | 15.48 |
| Residence | Urban | 2351(21.83) | 22.23 |
|  | Rural | 8421(78.17) | 77.77 |
| Marital Status | No | 8176(75.90) | 73.26 |
|  | Yes | 2596(24.10) | 26.74 |
| Gender | Male | 3774(35.04) | 30.02 |
|  | Female | 6998(64.96) | 69.98 |
| Gender–Marital types | Married Female | 2596(24.10) | 26.74 |
|  | Unmarried Female | 4402(40.87) | 43.24 |
|  | Unmarried Male | 3774(35.04) | 30.02 |
| Use Internet Device | No | 10123(93.98) | 92.61 |
|  | Yes | 649(6.02) | 7.39 |
| Wealth Quantile | Poorest | 1291(15.79) | 14.95 |
|  | Poorer | 1720(21.04) | 21.34 |
|  | Middle | 1751(21.42) | 21.20 |
|  | Richer | 1717(21.00) | 20.89 |
|  | Richest | 1697(20.76) | 21.61 |
| Currently Attend School / College | No | 3418(37.81) | 37.31 |
|  | Yes | 5622(62.19) | 62.69 |
| Division | Barisal | 580(5.38) | 5.75 |
|  | Chittagong | 2564(23.80) | 19.12 |
|  | Dhaka | 2183(20.27) | 23.91 |
|  | Khulna | 930(8.63) | 9.58 |
|  | Mymensingh | 1376(12.77) | 13.10 |
|  | Rajshahi | 1239(11.50) | 12.17 |
|  | Rangpur | 1020(9.47) | 7.45 |
|  | Sylhet | 880(8.17) | 8.92 |
