# Supplementary material for: Exploring the association between bullying and depression symptoms among adolescents in Bangladesh: Findings from nationally representative survey
Source: PLOS Ment Health. 2026 Oct 28;3(10):e0000722. doi: 10.1371/journal.pmen.0000722 (PMC13716191; doi:10.1371/journal.pmen.0000722)
Supplement: Peer review history

# Author comment: Author response to Decision Letter 0

**DOI:** 10.1371/journal.pmen.0000722.r001

12 Mar 2026

Attachment Submitted filename: Rebuttal_Adolescent_MentalHealth_Bullying (1).pdf

# Peer review: Decision Letter 0

**DOI:** 10.1371/journal.pmen.0000722.r002

**Authors:** Helena R. Slobodskaya

6 May 2026

PMEN-D-26-00157

Exploring the Association Between Bullying and Mental Well-being Among Adolescents in Bangladesh: Findings from Nationally Representative Survey

PLOS Mental Health

Dear Dr. Amin,

Thank you for submitting your manuscript to PLOS Mental Health. After careful consideration, we feel that it has merit but does not fully meet PLOS Mental Health’s publication criteria as it currently stands. Therefore, we invite you to submit a revised version of the manuscript that addresses the points raised during the review process.

Please submit your revised manuscript by Jul 05 2026 11:59PM. If you will need more time than this to complete your revisions, please reply to this message or contact the journal office at mentalhealth@plos.org. When you're ready to submit your revision, log on to https://www.editorialmanager.com/pmen/ and select the 'Submissions Needing Revision' folder to locate your manuscript file.

Please include the following items when submitting your revised manuscript:

- A letter that responds to each point raised by the editor and reviewer(s). You should upload this letter as a separate file labeled 'Response to Reviewers'.
- A marked-up copy of your manuscript that highlights changes made to the original version. You should upload this as a separate file labeled 'Revised Manuscript with Track Changes'.
- An unmarked version of your revised paper without tracked changes. You should upload this as a separate file labeled 'Manuscript'.

Guidelines for resubmitting your figure files are available below the reviewer comments at the end of this letter.

As the corresponding author, your ORCID iD is verified in the submission system and will appear in the published article. PLOS supports the use of ORCID, and we encourage all coauthors to register for an ORCID iD and use it as well. Please encourage your coauthors to verify their ORCID iD within the submission system before final acceptance, as unverified ORCID iDs will not appear in the published article. *Only*  the individual author can complete the verification step; PLOS staff *cannot*  verify ORCID iDs on behalf of authors.

We look forward to receiving your revised manuscript.

Kind regards,

Helena R. Slobodskaya, M.D., Ph.D., D.Sc.

Academic Editor

PLOS Mental Health

**Journal Requirements:**

1. Please note that PLOS Mental Health has specific guidelines on code sharing for submissions in which author-generated code underpins the findings in the manuscript. In these cases, we expect all author-generated code to be made available without restrictions upon publication of the work. Please review our guidelines at https://journals.plos.org/mentalhealth/s/materials-and-software-sharing#loc-sharing-code and ensure that your code is shared in a way that follows best practice and facilitates reproducibility and reuse.

2. Thank you for uploading your study's underlying data set. Unfortunately, the repository you have noted in your Data Availability statement does not qualify as an acceptable data repository according to PLOS's standards.

At this time, please upload the minimal data set necessary to replicate your study's findings to a stable, public repository (such as figshare or Dryad) and provide us with the relevant URLs, DOIs, or accession numbers that may be used to access these data. For a list of recommended repositories and additional information on PLOS standards for data deposition, please see https://journals.plos.org/plosone/s/recommended-repositories.

3. Some material included in your submission may be copyrighted. According to PLOS’s copyright policy, authors who use figures or other material (e.g., graphics, clipart, maps) from another author or copyright holder must demonstrate or obtain permission to publish this material under the Creative Commons Attribution 4.0 International (CC BY 4.0) License used by PLOS journals. Please closely review the details of PLOS’s copyright requirements here: PLOS Licenses and Copyright. If you need to request permissions from a copyright holder, you may use PLOS's Copyright Content Permission form.

Please respond directly to this email or email the journal office and provide any known details concerning your material's license terms and permissions required for reuse, even if you have not yet obtained copyright permissions or are unsure of your material's copyright compatibility.

Potential Copyright Issues:

a. S2 Fig: please (a) provide a direct link to the base layer of the map (i.e., the country or region border shape) and ensure this is also included in the figure legend; and (b) provide a link to the terms of use / license information for the base layer image or shapefile. We cannot publish proprietary or copyrighted maps (e.g. Google Maps, Mapquest) and the terms of use for your map base layer must be compatible with our CC-BY 4.0 license.

Note: if you created the map in a software program like R or ArcGIS, please locate and indicate the source of the basemap shapefile onto which data has been plotted.

If your map was obtained from a copyrighted source please amend the figure so that the base map used is from an openly available source. Alternatively, please provide explicit written permission from the copyright holder granting you the right to publish the material under our CC-BY 4.0 license.

Please note that the following CC BY licenses are compatible with PLOS license: CC BY 4.0, CC BY 2.0 and CC BY 3.0, meanwhile such licenses as CC BY-ND 3.0 and others are not compatible due to additional restrictions.

If you are unsure whether you can use a map or not, please do reach out and we will be able to help you. The following websites are good examples of where you can source open access or public domain maps:

* U.S. Geological Survey (USGS) - All maps are in the public domain. (http://www.usgs.gov)

* PlaniGlobe - All maps are published under a Creative Commons license so please cite “PlaniGlobe, http://www.planiglobe.com, CC BY 2.0” in the image credit after the caption. (http://www.planiglobe.com/?lang=enl)

* Natural Earth - All maps are public domain. (http://www.naturalearthdata.com/about/terms-of-use/)

If the reviewer comments include a recommendation to cite specific previously published works, please review and evaluate these publications to determine whether they are relevant and should be cited. There is no requirement to cite these works unless the editor has indicated otherwise.

**Additional Editor Comments (if provided):**

Two experts in this area have now reviewed your manuscript and I independently read and evaluated the manuscript as well. A number of strengths were noted about the manuscript, although significant concerns were identified as well. Thus, I cannot recommend that your manuscript be accepted for publication in PLOS Mental Health at this time. However, if you feel that you can adequately address the concerns raised by myself and the reviewers, I invite to you to submit a substantially revised manuscript for further consideration.

The reviewers comments and suggestions are clear and direct. In addition, I specifically encourage you to consider your statistical approach.

First, the division of the sample into subsamples based on gender and marital status (married females, unmarried females and unmarried males) seems rather unsubstantiated and serves as the manuscript main weakness. I would suggest to include adolescent gender and marital status as explanatory variables in addition to age, residence, wealth and school/college attendance. It would be helpful to see the gender and marital status distribution in Table 1 along with gender and marital status differences in Table 2. Figures 1 and 2, by contrast, do not seem to be informative and should be removed.

I agree with Reviewer 2 that it is important to consider multiple types of bullying and to examine the effects of bullying in more detail. It might be helpful to create a composite measure of bullying and to compare the effects of specific types of bullying and the composite measure.

It is unclear from the Statistical Analysis subsection and the Results whether the coefficients in Table 3 present results from a series of linear mixed models, each with one predictor variable, or results from one linear mixed model with multiple predictors.

I strongly recommend to examine all possible explanatory variables in one regression model including: first, main effects of bullying, socioeconomic and demographic variables; and, second, bivariate interactions of bullying, socioeconomic and demographic variables. I agree with Reviewer 1 that interaction tests are crucial for understanding how the association of bullying with depressive symptoms varies across socio-demographic groups.

Finally, I would suggest to present comparisons between 2- and 3-level mixed models in the preliminary analysis subsection. Although this might be valuable information, the focus of this study is on the relations of bullying and depressive symptoms in adolescents from different socio-demographic groups.

[Note: HTML markup is below. Please do not edit.]

Reviewers' comments:

Reviewer's Responses to Questions

**Comments to the Author**

1. Does this manuscript meet PLOS Mental Health’s publication criteria?

Reviewer #1: No

Reviewer #2: Partly

**********

2. Has the statistical analysis been performed appropriately and rigorously?

Reviewer #1: Yes

Reviewer #2: No

**********

3. Have the authors made all data underlying the findings in their manuscript fully available (please refer to the Data Availability Statement at the start of the manuscript PDF file)?

The PLOS Data policy

Reviewer #1: Yes

Reviewer #2: Yes

**********

4. Is the manuscript presented in an intelligible fashion and written in standard English?

Reviewer #1: No

Reviewer #2: Yes

**********

**Reviewer #1:**  Thank you for the opportunity to review this manuscript. The topic is important, and I appreciate the authors’ effort to examine bullying and depressive symptoms among adolescents in a lower-income setting using nationally representative survey data. This is a relevant public health question, and evidence from Bangladesh is valuable. However, the manuscript requires major revision. In its current form, I do not believe it is suitable for acceptance because of substantial problems in conceptual framing, methods reporting, results presentation, and interpretation.

Introduction

The introduction requires substantial restructuring and editing. At present, it is too broad, repetitive, and conceptually inconsistent. The manuscript moves back and forth between “mental health,” “mental well-being,” “depression,” and even broader psychological outcomes, but the actual outcome analyzed is a PHQ-9 depression score. The introduction should therefore be rewritten around a much more focused question: bullying and depressive symptoms among Bangladeshi adolescents. The long global overview is not well integrated into the study rationale, and the Bangladesh-specific gap appears too late.

There are also major writing problems. Some passages are redundant or awkwardly phrased, and several sentences require editing for clarity and grammar. A clear example is the repeated England school-survey sentence in the physical bullying section, where essentially the same point is stated twice consecutively. The subsection “In Present Study” is also poorly written and reads as a patchwork summary rather than a coherent rationale.

The novelty and gap statements are overstated. The authors claim that “no research in Bangladesh” has examined these associations, yet the introduction itself cites Bangladesh studies linking cyberbullying, violence, and depression. A more defensible claim would be that this study integrates multiple bullying forms in one national analysis and examines available subgroups within the BAHWS dataset. The current framing overstates what is new.

The hypotheses also need revision. They are framed around “mental health,” although the modeled outcome is depression. They are also largely descriptive and are not tightly linked to a conceptual framework. I would recommend either simplifying the aims or reformulating the hypotheses so they directly match the measured outcome and planned subgroup comparisons.

Methods

The most important conceptual problem in the methods is the outcome definition. The manuscript repeatedly calls the outcome “mental wellbeing,” but the outcome is a summed modified PHQ-9 score. That is a measure of depressive symptom severity, not general mental well-being. This needs to be corrected throughout the manuscript. In addition, the authors state that they used a “modified” PHQ-9, but they do not explain what was modified, whether the adapted version was validated in this setting, or what reliability was observed in the analytic sample.

The sample selection also requires much clearer reporting. The survey initially identified 18,249 adolescents, but only 9,116 were included because they had “plausible information” for calculating the outcome. Excluding roughly half of the identified sample is not a minor issue. The authors need to explain exactly why so many participants were excluded, provide a missing-data description, and discuss the possibility of selection bias. A comparison between included and excluded cases would strengthen the paper substantially.

There is also an important mismatch in time frames: bullying exposures were assessed over the previous 12 months, whereas depressive symptoms were assessed over the previous 2 weeks. This does not invalidate the analysis, but it is a key design limitation and should be acknowledged explicitly, because it weakens temporal interpretation.

The statistical analysis section needs clarification. The authors state that sample weights were applied and that a multilevel linear mixed model was used, but it is not clearly explained how the complex survey design and weighting were incorporated into the multilevel analysis. For nationally representative survey data, this point is methodologically important. In addition, the choice of a linear mixed model for a bounded symptom score should be justified more explicitly, and model diagnostics or sensitivity analyses would be helpful.

Finally, the subgroup structure needs a clearer methodological rationale. The manuscript analyzes ever-married females, unmarried females, and unmarried males, while acknowledging that married adolescent boys were unavailable in the dataset. That limitation is noted later, but the methods should explain the subgroup strategy more transparently at the outset.

Results

The results section contains reporting problems that need correction before the findings can be evaluated with confidence. Most notably, the text describing Table 3 states that physical bullying, verbal bullying, and cyberbullying were associated with depression by “2.11, 2.11, and 2.11,” whereas Table 3 shows coefficients of 1.12, 1.30, and 2.11. This is a direct inconsistency between the text and the table.

There are also typographical or data-presentation errors in the descriptive tables. For example, in Table 1 the weighted percentage for age 17 appears as “2074,” which is presumably 20.74. Errors of this kind undermine confidence in the rigor of the results section and should be carefully corrected throughout.

The subgroup results are potentially interesting, but the authors overinterpret between-group differences. They describe some bullying forms as having larger effects in certain subgroups, yet I do not see formal interaction tests showing that these differences between ever-married females, unmarried females, and unmarried males are statistically different from one another. Without interaction analyses, these comparisons should be described more cautiously.

The multilevel model results also need to be interpreted more carefully. The 3-level model shows somewhat better fit than the 2-level model, but the improvement appears modest, and the ICC changes only from 0.030 to 0.033. This supports the use of a multilevel approach, but not a strong claim that the model meaningfully transformed the substantive conclusions.

Discussion

The discussion substantially overreaches the data. The authors frequently revert to “mental health” and “mental well-being” language, even though the outcome modeled is depression. They also refer to anxiety, suicidal ideation, and broader psychosocial consequences that were not directly analyzed in this study. The discussion should remain centered on what was actually measured: depressive symptoms.

Several interpretations are not fully supported by the presented results. For example, the discussion states that there were significant associations with “gender,” but gender is not presented as a direct covariate in the all-adolescent model shown in Table 3; rather, the paper presents separate subgroup analyses. Similarly, the discussion describes a “U-shaped” relationship between wealth and depression, but in the adjusted model the richest group is not significantly different from the poorest group. That interpretation should therefore be toned down.

The statement that cyberbullying was the “most damaging” form of bullying is also too strong for a cross-sectional analysis. The data support that cyberbullying had the strongest observed association with depressive symptoms in these models, but “most damaging” implies a causal and comparative claim that the design cannot establish. The same issue appears again in the conclusions.

The novelty claim is again overstated in the discussion. The manuscript presents the multilevel modeling approach as a unique contribution, but the model fit statistics suggest only a marginal gain. The paper may have incremental value as a national Bangladesh analysis, but it should not be framed as methodologically novel on the basis of this modeling choice alone.

The limitations section is incomplete. In addition to the cross-sectional nature of the data and self-report bias, the authors should explicitly discuss the large reduction from 18,249 to 9,116 participants, the mismatch between bullying and depression time windows, and the lack of explicit suicide-specific measurement despite occasional discussion language that drifts in that direction.

**Reviewer #2:**  Introduction

(1) In the Introduction, the term “bullying” and “harassment” are used interchangeably. The authors need to define each behavior.

(2) More information regarding with marital status and education in Bangladesh is needed such as average age of marriage, the rate of the enrollment for higher education such as college or university. Cultural background affects the bullying behavior and depression.

(3) What percentage of adolescents who own internet devices? Information regarding with smartphones, PCs and other devices should be mentioned.

(4) On line57 to 78 should be more organized. In line 57 to 67, many countries and many regions are written. Need to be written separately and need to be structured.

(5) On 146 to 157, the description needs to be divided in paragraphs.

Method

The method section needs to be more structured and revised.

(1) In the survey, how the victimizations of bullying were assessed? Since the percentage of the bullying depend much on the expression of the question, the authors need to clarify the questions. Is the frequency assessed? Intensity might affect the mental health. More precise information is needed.

(2) Some of the adolescents experience multiple types of bullying. The authors need to conduct further analysis and clarify the following results; percentage of the adolescents who have been victimized of multiple types of bullying, how the victimization of multiple types of victim associate with the degree of depression.

Discussion

(1) Some of the descriptions are arbitrary. For example, on line 342 the authors state as follows; “Our findings confirmed that bullying victimization is strongly associated with poor adolescent mental health outcomes, particularly depression.” How did the authors conclude that the association is “strong”? Some of the CIs are not as high as could be judged as “strong”, On line 354, the authors state that “Adolescents exposed to bullying consistently reported higher depression levels, a result in line with international evidence that highlights bullying as a major risk factor for adolescent mental health problems.” However, whether the adolescents exposed to bullying “consistently” is not assessed in the questionnaire. At least, it is not written in the method.

(2) The paragraph of the Discussion part is too long. The discussion of the two hypotheses is included in one paragraph2. It should be written separately.

(3) On Line 343-344, the authors state “. Our findings confirmed that bullying victimization is strongly associated with poor adolescent mental health outcomes, particularly depression.” However, the authors did not examine mental health outcomes other than depression. More precise description is needed.

(4) Line 393 to 410 is the repletion of the Results. The insights from the results should be described in the Discussion.

**********

what does this mean?). If published, this will include your full peer review and any attached files.

**Do you want your identity to be public for this peer review?**  If you choose “no”, your identity will remain anonymous but your review may still be made public.

For information about this choice, including consent withdrawal, please see our Privacy Policy

Reviewer #1: No

Reviewer #2: No

**********

[NOTE: If reviewer comments were submitted as an attachment file, they will be attached to this email and accessible via the submission site. Please log into your account, locate the manuscript record, and check for the action link "View Attachments". If this link does not appear, there are no attachment files.]

**Figure Resubmissions:**

# Author comment: Author response to Decision Letter 1

**DOI:** 10.1371/journal.pmen.0000722.r003

5 Jul 2026

Attachment Submitted filename: Rebuttal__AdolescentMentalHealth_V1.pdf

# Peer review: Decision Letter 1

**DOI:** 10.1371/journal.pmen.0000722.r004

**Authors:** Helena R. Slobodskaya

17 Aug 2026

PMEN-D-26-00157R1

Exploring the Association Between Bullying and Depression Symptoms Among Adolescents in Bangladesh: Findings from Nationally Representative Survey

PLOS Mental Health

Dear Dr. Amin,

Thank you for submitting your manuscript to PLOS Mental Health. After careful consideration, we feel that it has merit but does not fully meet PLOS Mental Health’s publication criteria as it currently stands. Therefore, we invite you to submit a revised version of the manuscript that addresses the points raised during the review process.

The same two experts have now reviewed the revised manuscript and I independently evaluated the revision as well. As you can see, despite important strengths, significant concerns remain as well. It is not common to ask for a second, major, revision, but I feel I cannot accept the manuscript for publication in its present form. However, if you wish to undertake major revisions and re-submit addressing the concerns raised by myself and the reviewers, I would be willing to reconsider the manuscript for publication.

I believe you will find the reviewers' comments quite clear. I offer a number of points to consider in a potential revision, as well.

1. Linear mixed models: Please, indicate in Statistical Analysis if you tested two models in the total sample: 1) with the three bullying variables and 2) with the composite bullying. It is not clear whether the same two models (without gender and marital status) were also tested in the three subgroups and if not, why.

2. Results, 1^st^ para and Table 1: Please check the figures for gender-by-marital status groups, they do not sum up correctly: if there were 2470 (27%) married females and no married males, why total number of married participants was 6646 (73%)? Reviewer 1 noted other substantive discrepancies that need to be addressed.

3. Regarding comparisons between 2- and 3-level mixed models, I was not able to find the Preliminary analysis subsection in the manuscript.

Please submit your revised manuscript by Oct 16 2026 11:59PM. If you will need more time than this to complete your revisions, please reply to this message or contact the journal office at mentalhealth@plos.org. When you're ready to submit your revision, log on to https://www.editorialmanager.com/pmen/ and select the 'Submissions Needing Revision' folder to locate your manuscript file.

Please include the following items when submitting your revised manuscript:

- A letter that responds to each point raised by the editor and reviewer(s). You should upload this letter as a separate file labeled 'Response to Reviewers'.
- A marked-up copy of your manuscript that highlights changes made to the original version. You should upload this as a separate file labeled 'Revised Manuscript with Track Changes'.
- An unmarked version of your revised paper without tracked changes. You should upload this as a separate file labeled 'Manuscript'.

Guidelines for resubmitting your figure files are available below the reviewer comments at the end of this letter.

As the corresponding author, your ORCID iD is verified in the submission system and will appear in the published article. PLOS supports the use of ORCID, and we encourage all coauthors to register for an ORCID iD and use it as well. Please encourage your coauthors to verify their ORCID iD within the submission system before final acceptance, as unverified ORCID iDs will not appear in the published article. *Only*  the individual author can complete the verification step; PLOS staff *cannot*  verify ORCID iDs on behalf of authors.

We look forward to receiving your revised manuscript.

Kind regards,

Helena R. Slobodskaya, M.D., Ph.D., D.Sc.

Academic Editor

PLOS Mental Health

**Journal Requirements:**

If the reviewer comments include a recommendation to cite specific previously published works, please review and evaluate these publications to determine whether they are relevant and should be cited. There is no requirement to cite these works unless the editor has indicated otherwise.

**Additional Editor Comments (if provided):**

[Note: HTML markup is below. Please do not edit.]

**Reviewers' comments:**

Reviewer's Responses to Questions

**Comments to the Author**

Reviewer #1: (No Response)

Reviewer #2: All comments have been addressed

**********

publication criteria?

Reviewer #1: Yes

Reviewer #2: Partly

**********

3. Has the statistical analysis been performed appropriately and rigorously?

Reviewer #1: No

Reviewer #2: Yes

**********

4. Have the authors made all data underlying the findings in their manuscript fully available (please refer to the Data Availability Statement at the start of the manuscript PDF file)?

The PLOS Data policy

Reviewer #1: No

Reviewer #2: Yes

**********

5. Is the manuscript presented in an intelligible fashion and written in standard English?

Reviewer #1: Yes

Reviewer #2: Yes

**********

**Reviewer #1:**  The authors use the Bangladesh Adolescent Health and Wellbeing Survey 2019-20 to examine associations between physical, verbal, and cyber bullying victimisation and PHQ-9 depressive symptom scores among adolescents aged 15-19, using a three-level linear mixed model. They report independent associations for each bullying form, a gender interaction for physical bullying, and a dose-response gradient across a composite count of bullying types. They also report subgroup analyses across the three populations the survey covers, ever-married females, unmarried females, and unmarried males, and conclude that these support their second hypothesis.

General assessment

The question is worth asking and the dataset is the right one. Nearly the entire evidence base on bullying and adolescent depression comes from school-based samples in high-income settings, where "adolescent" is functionally synonymous with "student" and "peer" with "classmate." Bangladesh is one of the few contexts where a national survey can separate those, because a large share of adolescents, overwhelmingly girls, leave school and enter a marital household during the exposure window. That makes this a rare opportunity to ask whether the bullying-depression association is specific to peer-group processes or reflects interpersonal victimisation more broadly, and whether early marriage changes the exposure setting, the vulnerability, or both. My concern is that the paper's distinctive contribution sits in its least developed analyses, and that several reporting problems currently block assessment of the core claims.

Major comments

1. The operationalization of “bullying” is a central concern: The measures appear to capture any physical violence, verbal/social mistreatment, or online harassment by “anyone,” and a single occurrence appears sufficient for classification. This does not necessarily meet the usual definition of bullying involving repetition and a power imbalance. The authors should either justify these measures as bullying or, more appropriately, relabel the exposures as violence/harassment throughout and reconsider the framing of the paper.

2. The large reduction in the analytic sample remains insufficiently resolved: Only 9,116 of 19,888 identified adolescents contributed complete PHQ-9 data. Given that different questionnaire modules were administered across cluster types, the authors should clarify whether this reflects true missingness or the survey design itself. This distinction is critical for selection bias, weighting, and the claim that the analytic results are nationally representative.

3. There are several substantive inconsistencies across the reported results that require a complete audit: For example, Table 1 reports 72.75% as married, whereas the gender–marital categories indicate that 27.25% were married females; the abstract describes ever-married status as associated with higher depressive symptoms although marital status is non-significant in the adjusted model; and the abstract reports a significant verbal-bullying × gender interaction whereas Table 4 reports this interaction as non-significant. These discrepancies substantially undermine confidence in the current results and should be reconciled throughout.

4. The claims regarding differences by sex and marital-status group exceed the formal analyses presented: Separate significant associations within ever-married females, unmarried females, and unmarried males do not demonstrate that associations differ between these groups. If variation by marital status is a central hypothesis, this should be tested formally through appropriate interaction terms; otherwise, the subgroup findings should remain descriptive/exploratory and conclusions regarding between-group differences should be substantially tempered.

**Reviewer #2:**  Review

The manuscript is revised. However, it does not seem to be adequate.

(1) The authors stated as follows: “Although the legal minimum age of marriage is 18

years for females and 21 years for males, the median age at first marriage among women remains 16.9 years, and approximately 51.4% of women aged 20–24 years were 84 married before the age of 18, indicating that child marriage remains common despite legal 85 restrictions.”

It is restricted by the law, how the adolescents get married?

What is the definition of marriage in this paper? Explanations cause confusion, thus the authors need to add more explanation on the cultural and societal background. Moreover, the authors need to elucidate socioeconomical characteristics that early-married adolescents have. These background associate with the victimization.

(2) The authors added analysis shown in Table5 based on the reviewer’s comment. The authors should include the hypothesis regarding with this analysis in the Introduction part. Moreover, the authors need to describe the effect of victimization of multiple types of bullying in Discussion part.

**********

what does this mean?). If published, this will include your full peer review and any attached files.

**Do you want your identity to be public for this peer review?**  If you choose “no”, your identity will remain anonymous but your review may still be made public.

For information about this choice, including consent withdrawal, please see our Privacy Policy

Reviewer #1: No

Reviewer #2: No

**********

[NOTE: If reviewer comments were submitted as an attachment file, they will be attached to this email and accessible via the submission site. Please log into your account, locate the manuscript record, and check for the action link "View Attachments". If this link does not appear, there are no attachment files.]

**Figure Resubmissions:**

# Author comment: Author response to Decision Letter 2

**DOI:** 10.1371/journal.pmen.0000722.r005

24 Aug 2026

Attachment Submitted filename: Rebuttal__AdolescentMentalHealth.pdf

# Peer review: Decision Letter 2

**DOI:** 10.1371/journal.pmen.0000722.r006

**Authors:** Helena R. Slobodskaya

14 Sep 2026

PMEN-D-26-00157R2

Exploring the Association Between Bullying and Depression Symptoms Among Adolescents in Bangladesh: Findings from Nationally Representative Survey

PLOS Mental Health

Dear Dr. Amin,

Thank you for submitting your manuscript to PLOS Mental Health. After careful consideration, we feel that it has merit but does not fully meet PLOS Mental Health’s publication criteria as it currently stands. Therefore, we invite you to submit a revised version of the manuscript that addresses the points raised during the review process.

The same two experts have now reviewed the revised manuscript and I independently evaluated the 2^nd^ revision as well. As you can see, although the Reviewers acknowledge that all comments have been addressed, they differ in their recommendations. I also share some of the concerns that were raised in the earlier decision. Therefore, I would like to ask you to read these comments and get the paper back to me after appropriate revisions are made.

1. Linear mixed models. I was not able to find the description of your analytical strategy and its rationale on Pages 09-10, Lines 248–251. It is still unclear why the model with the composite bullying was not tested in the three subgroups. Please explain this in Statistical Analysis subsection.

2. Please address Reviewer 2 concern about the reliability of the data on married adolescents. It would be helpful to describe how marriage procedures for those under statutory minimum age differ from the standard marriage procedures. The possibility of mixing domestic violence with bullying should also be addressed and clearly acknowledged as a limitation.

3. In response to Reviewer 2 comment # 1 about marriage, you added a new paragraph to the Introduction (Page 4-5, lines 84-101). However, this text largely repeats the next paragraph on Page 5, lines 102-116. Please, edit the text carefully to avoid repetitions and to improve the precision and coherence of the manuscript.

Please submit your revised manuscript by Oct 14 2026 11:59PM. If you will need more time than this to complete your revisions, please reply to this message or contact the journal office at mentalhealth@plos.org. When you're ready to submit your revision, log on to https://www.editorialmanager.com/pmen/ and select the 'Submissions Needing Revision' folder to locate your manuscript file.

Please include the following items when submitting your revised manuscript:

- A letter that responds to each point raised by the editor and reviewer(s). You should upload this letter as a separate file labeled 'Response to Reviewers'.
- A marked-up copy of your manuscript that highlights changes made to the original version. You should upload this as a separate file labeled 'Revised Manuscript with Track Changes'.
- An unmarked version of your revised paper without tracked changes. You should upload this as a separate file labeled 'Manuscript'.

Guidelines for resubmitting your figure files are available below the reviewer comments at the end of this letter.

As the corresponding author, your ORCID iD is verified in the submission system and will appear in the published article. PLOS supports the use of ORCID, and we encourage all coauthors to register for an ORCID iD and use it as well. Please encourage your coauthors to verify their ORCID iD within the submission system before final acceptance, as unverified ORCID iDs will not appear in the published article. *Only*  the individual author can complete the verification step; PLOS staff *cannot*  verify ORCID iDs on behalf of authors.

We look forward to receiving your revised manuscript.

Kind regards,

Helena R. Slobodskaya, M.D., Ph.D., D.Sc.

Academic Editor

PLOS Mental Health

Journal Requirements:

If the reviewer comments include a recommendation to cite specific previously published works, please review and evaluate these publications to determine whether they are relevant and should be cited. There is no requirement to cite these works unless the editor has indicated otherwise.

Please review your reference list to ensure that it is complete and correct. If you have cited papers that have been retracted, please include the rationale for doing so in the manuscript text, or remove these references and replace them with relevant current references. Any changes to the reference list should be mentioned in the rebuttal letter that accompanies your revised manuscript. If you need to cite a retracted article, indicate the article’s retracted status in the References list and also include a citation and full reference for the retraction notice.

Additional Editor Comments (if provided):

[Note: HTML markup is below. Please do not edit.]

Reviewers' comments:

Reviewer's Responses to Questions

**Comments to the Author**

Reviewer #1: All comments have been addressed

Reviewer #2: All comments have been addressed

**********

publication criteria?

Reviewer #1: Yes

Reviewer #2: No

**********

3. Has the statistical analysis been performed appropriately and rigorously?

Reviewer #1: Yes

Reviewer #2: Yes

**********

4. Have the authors made all data underlying the findings in their manuscript fully available (please refer to the Data Availability Statement at the start of the manuscript PDF file)?

The PLOS Data policy

Reviewer #1: (No Response)

Reviewer #2: Yes

**********

5. Is the manuscript presented in an intelligible fashion and written in standard English?

Reviewer #1: Yes

Reviewer #2: Yes

**********

**Review Comments to the Author**

Reviewer #1: no further comments

Reviewer #2: Marriage is governed by law. Although the law prohibits women under the age of 18 from marrying, it is not clearly specified how marriage procedures are carried out in such cases.This is an important point that affects the reliability of the data, but the authors do not explain it.

Moreover, in the survey, by whom the respondents are bullied. If any of the respondents are married, domestic violence may be included in the "bullied"participants. For this reason, the data can be considered unreliable.

**********

what does this mean?). If published, this will include your full peer review and any attached files.

**Do you want your identity to be public for this peer review?**  If you choose “no”, your identity will remain anonymous but your review may still be made public.

For information about this choice, including consent withdrawal, please see our Privacy Policy

Reviewer #1: No

Reviewer #2: No

**********

[NOTE: If reviewer comments were submitted as an attachment file, they will be attached to this email and accessible via the submission site. Please log into your account, locate the manuscript record, and check for the action link "View Attachments". If this link does not appear, there are no attachment files.]

# Author comment: Author response to Decision Letter 3

**DOI:** 10.1371/journal.pmen.0000722.r007

26 Sep 2026

Attachment Submitted filename: Rebuttal__AdolescentMentalHealth_auresp_3.pdf

# Editor report: Decision Letter 3

**DOI:** 10.1371/journal.pmen.0000722.r008

**Authors:** Helena R. Slobodskaya

5 Oct 2026

PMEN-D-26-00157R3

Exploring the Association Between Bullying and Depression Symptoms Among Adolescents in Bangladesh: Findings from Nationally Representative Survey

PLOS Mental Health

Dear Dr. Amin,

Thank you for submitting your manuscript to PLOS Mental Health. After careful consideration, we feel that it has merit but does not fully meet PLOS Mental Health’s publication criteria as it currently stands. Therefore, we invite you to submit a revised version of the manuscript that addresses the points raised during the review process.

I believe all main issues have been adequately resolved. However, I still feel that the paper is difficult to get through. Therefore, I would like to ask for a structural edit to avoid repetitions and achieve greater clarity. Below are some of the suggestions.

1. Please define the abbreviation BAHWS when first introduced on p. 4, line 90 (Adolescent Mental Wellbeing_Marked_RA_V4_with track changes).

2. The statutory minimum age for marriage is presented twice: in the Introduction on p. 4, line 86, and in the Data Sources subsection on p. 7, line 179. Please remove the repetition.

3. The BAHWS is also described twice: in the Introduction on p. 5, lines 130-133, and in the Data Sources subsection on p. 7, lines 164-170. I would suggest to present all details of the study design in the Data Sources subsection. In the Introduction the Authors could briefly indicate the hierarchical nature of the survey data.

4. A linear mixed model is described in the first paragraph of Statistical Analysis on p.10, and then in a separate subsection on pp. 12-13. I would suggest to place the Linear Mixed Model subsection after the sentence "Since the study employed a multilevel data structure, we utilized a linear mixed model to identify the significant determinants that addressed the within-group correlation [31,32]". It would be helpful to place the text about criteria used for model selection (on p. 10, lines 285-288) at the end of the Linear Mixed Model subsection.

5. In Statistical Analysis, subgroup analyses is presented four times: at the end of the first paragraph on p. 10, lines 83-84; in the second paragraph on p. 11, lines 294-300 and 305-307, and in the third paragraph on pp. 11-12, lines 318-326. I would suggest to describe each of the the three models tested after the subsection on the Linear Mixed Model in the same order as they are presented in the Results: (1) the main model with three bullying variables in the total sample, (2) the main model with three bullying variables in the three subgroups and (3) the additional model with composite bullying in the total sample.

6. Please explain how the coefficients for each value of the composite bullying measure (Table 5) were calculated.

7. In the Results, the selection of the multilevel model subsection is presented after the subsection "Descriptive characteristics and distribution of depressive symptoms". Therefore, it would be helpful to remove "Preliminary analysis" from the subtitle.

8. I would suggest to describe strengths and limitations in a separate subsection after Discussion and before the Conclusions.

Please submit your revised manuscript by Nov 04 2026 11:59PM. If you will need more time than this to complete your revisions, please reply to this message or contact the journal office at mentalhealth@plos.org. When you're ready to submit your revision, log on to https://www.editorialmanager.com/pmen/ and select the 'Submissions Needing Revision' folder to locate your manuscript file.

Please include the following items when submitting your revised manuscript:

- A letter that responds to each point raised by the editor and reviewer(s). You should upload this letter as a separate file labeled 'Response to Reviewers'.
- A marked-up copy of your manuscript that highlights changes made to the original version. You should upload this as a separate file labeled 'Revised Manuscript with Track Changes'.
- An unmarked version of your revised paper without tracked changes. You should upload this as a separate file labeled 'Manuscript'.

Guidelines for resubmitting your figure files are available below the reviewer comments at the end of this letter.

As the corresponding author, your ORCID iD is verified in the submission system and will appear in the published article. PLOS supports the use of ORCID, and we encourage all coauthors to register for an ORCID iD and use it as well. Please encourage your coauthors to verify their ORCID iD within the submission system before final acceptance, as unverified ORCID iDs will not appear in the published article. *Only*  the individual author can complete the verification step; PLOS staff *cannot*  verify ORCID iDs on behalf of authors.

We look forward to receiving your revised manuscript.

Kind regards,

Helena R. Slobodskaya, M.D., Ph.D., D.Sc.

Academic Editor

PLOS Mental Health

Journal Requirements:

If the reviewer comments include a recommendation to cite specific previously published works, please review and evaluate these publications to determine whether they are relevant and should be cited. There is no requirement to cite these works unless the editor has indicated otherwise.

Please review your reference list to ensure that it is complete and correct. If you have cited papers that have been retracted, please include the rationale for doing so in the manuscript text, or remove these references and replace them with relevant current references. Any changes to the reference list should be mentioned in the rebuttal letter that accompanies your revised manuscript. If you need to cite a retracted article, indicate the article’s retracted status in the References list and also include a citation and full reference for the retraction notice.

Additional Editor Comments (if provided):

[Note: HTML markup is below. Please do not edit.]

Reviewers' comments:

[NOTE: If reviewer comments were submitted as an attachment file, they will be attached to this email and accessible via the submission site. Please log into your account, locate the manuscript record, and check for the action link "View Attachments". If this link does not appear, there are no attachment files.]

# Author comment: Author response to Decision Letter 4

**DOI:** 10.1371/journal.pmen.0000722.r009

5 Oct 2026

Attachment Submitted filename: Rebuttal__AdolescentMentalHealth_auresp_4.pdf

# Editor report: Decision Letter 4

**DOI:** 10.1371/journal.pmen.0000722.r010

**Authors:** Helena R. Slobodskaya

8 Oct 2026

Exploring the Association Between Bullying and Depression Symptoms Among Adolescents in Bangladesh: Findings from Nationally Representative Survey

PMEN-D-26-00157R4

Dear Mr. Amin,

We are pleased to inform you that your manuscript 'Exploring the Association Between Bullying and Depression Symptoms Among Adolescents in Bangladesh: Findings from Nationally Representative Survey' has been provisionally accepted for publication in PLOS Mental Health.

Before your manuscript can be formally accepted you will need to complete some formatting changes, which you will receive in a follow up email. A member of our team will be in touch with a set of requests.

Please note that your manuscript will not be scheduled for publication until you have made the required changes, so a swift response is appreciated.

IMPORTANT: The editorial review process is now complete. PLOS will only permit corrections to spelling, formatting or significant scientific errors from this point onwards. Requests for major changes, or any which affect the scientific understanding of your work, will cause delays to the publication date of your manuscript.

If your institution or institutions have a press office, please notify them about your upcoming paper to help maximize its impact. If they'll be preparing press materials, please inform our press team as soon as possible -- no later than 48 hours after receiving the formal acceptance. Your manuscript will remain under strict press embargo until 2 pm Eastern Time on the date of publication. For more information, please contact mentalhealth@plos.org.

Thank you again for supporting Open Access publishing; we are looking forward to publishing your work in PLOS Mental Health.

Best regards,

Helena R. Slobodskaya, M.D., Ph.D., D.Sc.

Academic Editor

PLOS Mental Health

***********************************************************

Reviewer Comments (if any, and for reference):
